# Cost-effectiveness Analysis of Nutrition Facts Added-Sugar Labeling and Obesity-Associated Cancer Rates in the US

**DOI:** 10.1001/jamanetworkopen.2021.7501

**Published:** 2021-04-27

**Authors:** Mengxi Du, Christina F. Griecci, Frederick F. Cudhea, Heesun Eom, David D. Kim, Parke Wilde, John B. Wong, Y. Claire Wang, Dominique S. Michaud, Dariush Mozaffarian, Fang Zhang

**Affiliations:** 1 Friedman School of Nutrition Science and Policy, Tufts University, Boston, Massachusetts; 2 Center for the Evaluation of Value and Risk in Health, Institute for Clinical Research and Health Policy Studies, Tufts Medical Center, Boston, Massachusetts; 3 Division of Clinical Decision Making, Tufts Medical Center, Boston, Massachusetts; 4 Department of Health Policy and Management, Mailman School of Public Health, Columbia University, New York, New York; 5 New York Academy of Medicine, New York; 6 Now with Division of Substance Abuse & Mental Health, Department of Health and Social Services, State of Delaware, Newcastle; 7 Department of Public Health and Community Medicine, School of Medicine, Tufts University, Boston, Massachusetts

## Abstract

**Importance:**

Obesity-associated cancer burdens are increasing in the US. Nutrition policies, such as the Nutrition Facts added-sugar labeling, may reduce obesity-associated cancer rates.

**Objective:**

To evaluate the cost-effectiveness of Nutrition Facts added-sugar labeling and obesity-associated cancer rates in the US.

**Design, Setting, and Participants:**

A probabilistic cohort state-transition model was used to conduct an economic evaluation of added-sugar labeling and 13 obesity-associated cancers among 235 million adults aged 20 years or older by age, sex, and race/ethnicity over a median follow-up of 34.4 years. Policy associations were considered in 2 scenarios: with consumer behaviors and with additional industry reformulation. The model integrated nationally representative population demographics, diet, and cancer statistics; associations of policy intervention with diet, diet change and body mass index, and body mass index with cancer risk; and policy and health-related costs from established sources. Data were analyzed from January 8, 2019, to May 6, 2020.

**Main Outcomes and Measures:**

Net costs and incremental cost-effectiveness ratio were estimated from societal and health care perspectives. Probabilistic sensitivity analyses incorporated uncertainty in input parameters and generated 95% uncertainty intervals (UIs).

**Results:**

Based on consumer behaviors, the policy was associated with a reduction of 30 000 (95% UI, 21 600-39 300) new cancer cases and 17 100 (95% UI, 12 400-22 700) cancer deaths, a gain of 116 000 (95% UI, 83 800-153 000) quality-adjusted life-years, and a saving of $1600 million (95% UI, $1190 million-$2030 million) in medical costs associated with cancer care among US adults over a lifetime. The policy was associated with a savings of $704 million (95% UI, $44.5 million-$1450 million) from the societal perspective and $1590 million (95% UI, $1180 million-$2020 million) from the health care perspective. Additional industry reformulation to reduce added-sugar amounts in packaged foods and beverages would double the impact. Greater health gains and cost savings were expected among young adults, women, and non-Hispanic Black individuals than other population subgroups.

**Conclusions and Relevance:**

These findings suggest that the added-sugar labeling is associated with reduced costs and lower rates of obesity-associated cancers. Policymakers may consider and prioritize nutrition policies for cancer prevention in the US.

## Key Points

**Question:**

What is the estimated association between added-sugar labeling and obesity-related cancer rates in the US?

**Findings:**

This economic evaluation of Nutrition Facts added-sugar labeling and obesity-related cancer rates estimated that implementing the policy was associated with a reduction of 30 000 new cancer cases, 17 100 cancer deaths, and $1600 million in medical costs among US adults over a lifetime. This policy would generate net savings of $704 million from a societal perspective and $1590 million from a health care perspective.

**Meaning:**

These model findings suggest added-sugar labeling would be associated with reduced costs and lower rates of obesity-related cancers.

## Teaser

This economic evaluation examines the association between the use of added-sugar labeling of food products in the US and the incidence of cancer from societal and health care perspectives.

## Introduction

Obesity is associated with an increased risk of 13 cancers.^1^ In the US, 631 000 individuals were diagnosed with obesity-associated cancers in 2014, accounting for 40% of all incident cancer cases.^2^ With the prevalence of overweight and obesity remaining high, obesity-associated cancers are contributing to substantial health and economic burdens in the US.

Evidence supports that high added sugar consumption contributes to obesity.^3^ Yet, US adults consume more than 14% of daily calories (approximately 300 kcals/d) from added sugars, exceeding the recommendation of the 2020-2025 Dietary Guidelines for Americans (<10% daily calories from added sugar).^4^ It was estimated that more than 3000 new cancer cases per year among US adults are attributable to increased added-sugar consumption from sugar-sweetened beverages alone^5^; decreasing added-sugar intake from all sources may be associated with reduced obesity-related cancer burdens.

In 2016, the US Food and Drug Administration (FDA) announced a mandatory labeling policy for all packaged foods and beverages to include the amount of added sugars on the Nutrition Facts label.^6^ This policy seeks to help consumers make informed food choices and reduce added sugar consumption.^6,7^ Manufacturers may also be motivated to reformulate sugar content in their products.^8,9^ However, the health benefit and economic association between the added-sugar labeling policy and obesity-associated cancer rates have not been evaluated. This study aimed to estimate the cost-effectiveness of the Nutrition Facts added-sugar labeling and obesity-associated cancer rates among US adults and further evaluate the association between implementing the policy and health disparities in population subgroups by age, sex, and race/ethnicity.

## Methods

### Study Overview

We used the Diet and Cancer Outcome Model,^10,11^ a probabilistic cohort state-transition model to perform an economic evaluation of the added-sugar labeling policy and obesity-related cancer rates among 235 million US adults aged 20 years or older over a simulated lifetime (eFigure 1 in the Supplement). The model integrated nationally representative population demographics, dietary intake, and cancer statistics; association estimates of policy intervention with diet, diet change with body mass index (BMI) (calculated as weight in kilograms divided by height in meters squared), and BMI with cancer risks; and policy and health-related costs from established sources^1,7,8,9,12,13,14,15,16,17,18,19,20,21,22,23,24^ (Table 1). Data were analyzed from January 8, 2019, to May 6, 2020. This study used deidentified national data sets and was exempted from review by the Tufts University Health Science Institutional Review Board because it did not involve human subjects as defined by the Department of Health and Human Services and US Food and Drug Administration. The study followed the Consolidated Health Economic Evaluation Reporting Standards (CHEERS) reporting guideline for economic evaluation studies.

**Table 1.** Key Input Parameters and Data Sources in the Dietary Cancer Outcome Model

Model input	Outcome	Estimates	Distribution	Comments	Data source
1. Simulated population	Population	Mean consumption of added sugars was 52.6 g/d from packaged foods and beverages (eTables 6-8 in the Supplement)	γ	Stratified by age, sex, race/ethnicity; baseline added-sugar intakes were estimated for each of the 32 subgroups	NHANES 2013-2016
2. Policy impact^a^					
Consumer behavior	Policy impact estimate	6.6% (95% CI, 4.4%-8.8%)	β	A 6.6% reduction in added-sugar consumption from packaged foods and beverages as a result of policy implementation; this was assumed as an 1-time impact	A meta-analysis of labeling interventions on reducing calorie intake^7^
Industry response	Policy impact estimate	8.25% (95% CI, 7.5%-9.0%)	β	Assumption: no reformulation in the 1st year of policy intervention; 7.5%-9.0% of the sugar-containing products are reformulated each of years 2-5 of the intervention to achieve a 25% reduction in added sugar content, resulting in a reduction of 8.25% of added-sugar intake associated with the policy intervention	FDA’s Regulatory Impact Analysis; UK sugar reduction strategy^8,9^
3. Association between change in added sugar intake (20 g/d) and change in BMI^a^	Diet-BMI association	Among individuals with BMI<25: 0.10 (95% CI, 0.05-0.15; BMI≥25: 0.23 (95% CI, 0.14-0.32)	Normal	Each 20-g/d reduction in added sugar leads to a 0.1-point reduction in BMI among healthy-weight individuals and a 0.23-point BMI reduction among overweight/obese individuals	A meta-analysis of prospective cohort studies^12^
				Assumption: an 8-oz sugar-sweetened beverage contains 20 g of added sugar based on NHANES; non–sugar-sweetened beverage added sugars has the same impact; the association between added sugar and BMI change would be maintained over a lifetime	
4. Association between BMI and cancer risks^a^	Cancer outcome	RR ranged from 1.05 to 1.50 (eTable 9 in the Supplement)	Log normal	BMI change and cancer incidence	Continuous Update Project conducted by the World Cancer Research Fund/American Institute for Cancer Research^13^
5. Cancer statistics^a^	Cancer incidence and survival	eAppendixes 1 and 2 in the Supplement	β	Stratified by age, sex, and race/ethnicity	NCI’s Surveillance, Epidemiology, and End Results Program Database; CDC’s National Program of Cancer Registries Database^1^
6. Health care–related costs^a^^,^^b^	Medical expenditures, productivity loss, and patient time costs	eTables 11 and 12 in the Supplement	γ	Stratified by age and sex	NCI’s Cancer Prevalence and Cost of Care Projections; published literature^14,17,18,19,20,21^
7. Policy costs^a^^,^^b^	For government and industry	eTable 2 in the Supplement	γ	Administration and monitoring costs for government; compliance and reformulation cost for industry	FDA’s budget report; Nutrition Review Project; and FDA’s RIA^8,15,16^
8. Health related quality of life^a^	For 13 types of cancers	Ranged from 0.64 to 0.86 (eTable 10 in the Supplement)	β	EQ-5D data from published literatures by cancer type^c^	Published literature^18,19,20,21,22,23,24^

Abbreviations: BMI, body mass index (calculated as weight in kilograms divided by height in meters squared); CDC, Centers for Disease Control and Prevention; FDA, US Food and Drug Administration; NCI, National Cancer Institute; NHANES, National Health and Nutrition Examination Survey; RR, relative risk.

^a^ Uncertainty distributions were incorporated in the probabilistic sensitivity analyses. Uncertainties in each parameter are presented in eTable 2 and eTables 6-12 in the Supplement.

^b^ If the original source did not provide uncertainty estimates, we assumed the SEs were 20% of the mean estimate to generate γ distribution.

^c^ EQ-5D is a standardized instrument developed by the EuroQol Group as a measure of health-related quality of life that can be used in a wide range of health conditions and treatments.

### Simulated US Population and Added Sugar Consumption

The 2013-2016 National Health and Nutrition Examination Survey data were used to simulate the US adult population aged 20 years or older in 32 subgroups by age (20-44, 45-54, 55-64, and ≥65 years), sex (men and women), and race/ethnicity (non-Hispanic White, non-Hispanic Black, Hispanic, and other) (eTable 6 and eTable 7 in the Supplement), assuming a closed cohort starting in 2015.

The mean added sugar consumption from packaged foods and beverages was estimated using data collected from participants with at least 1 valid 24-hour diet recall, stratified by 32 population subgroups (eTable 8 in the Supplement). Because added sugars are ubiquitously consumed in the US, to correct measurement errors associated with dietary intake estimated using 1 or 2 days of diet recalls, the amount-only National Cancer Institute method was applied to estimate the usual intake distribution of added sugars by fitting a linear regression on a transformed scale with person-specific outcomes.^25^ The prevalence of overweight or obesity in 32 subgroups was estimated based on the objectively measured BMI. The complex survey design was incorporated in all statistical analyses by using appropriate sampling weights, strata, and units to ensure the representativeness of the noninstitutionalized US adult population.

### Added Sugar Consumption and Obesity-Associated Cancer Risk

To estimate the relative risks of obesity-associated cancer risk with high added sugar consumption, we associated the multivariate-adjusted association of change in added sugar consumption (grams per day) with change in BMI (the diet-BMI associations) and the estimates of BMI and cancer risk (BMI-cancer relative risks). The diet-BMI estimates were obtained from a pooled analysis from 120 997 US men and women followed up over 12 to 20 years in 3 prospective cohort studies (Nurses’ Health Study, Nurses’ Health Study II, and Health Professionals Follow-up Study) (Table 1).^12,26^ The estimation accounted for potential calorie compensation (ie, compensating daily calories from other sources), facilitating more realistic and conservative estimates. The relative risks of cancer associated with each 5-point increase in BMI for 13 cancers were based on systematic reviews and meta-analyses of prospective cohort studies conducted by the World Cancer Research Fund/American Institute for Cancer Research Continuous Update Project and the International Agency for Research on Cancer (eTable 9 in the Supplement).^1,13^

### Cancer Incidence, Mortality, and Health-Related Quality of Life

Age-adjusted cancer incidence for 13 obesity-associated cancers in 2015 in each of the 32 population subgroups was obtained from the National Program of Cancer Registries and the Surveillance, Epidemiology, and End Results program.^27^ We projected the cancer incidence from 2015 to 2030 based on the incidence of cancer from 2006 to 2014 using the average annual percent change method (eAppendix 1 in the Supplement).^14^ We extracted and converted the 5-year relative survival rates for each cancer to an annual probability of death (eAppendix 2 and eTable 1 in the Supplement).^28^ Health-related quality of life was obtained from publications that reported the utility weights using EuroQol-5 Dimension for each cancer among the US patient population (eAppendix 3 and eTable 10 in the Supplement).

### Policy Association With Dietary Intake

Potential policy association was considered in 2 scenarios: consumers’ consumption of added sugars (ie, consumer behavior) and additional industry reformulation (ie, consumer behavior plus industry reformulation). The association with consumer behavior was obtained from a meta-analysis of food labeling interventions that reported a 6.6% (95% CI, 4.4%-8.8%) reduction in calories from menu calorie labeling.^7,29^ We assumed that the policy would result in a 1-time reduction in added sugar consumption during the first year of policy implementation, with no further increases or decreases thereafter. We further assumed no policy association with cancer risk within the first 5 years of implementation.

The association with industry reformation was estimated from the FDA’s regulatory impact analysis^8^ on the added-sugar labeling policy and the findings from the UK sugar reduction strategy.^9^ Based on these reports, we assumed no reformulation during the first year of implementation. In each of the subsequent 2 to 5 years, 7.5% to 9.0% of sugar-containing products would be reformulated to achieve a cumulative reduction of 25% in added sugar content of reformulated products,^8,9^ with no further changes thereafter. In total, an average 8.25% reduction in the added sugars would result from industry reformulation over 5 years (eAppendix 4 in the Supplement).

### Policy and Health-Related Costs

Policy costs were incorporated for all stakeholders, including costs for the government to administer, monitor, and evaluate the policy, costs for industry to comply with the policy, and additional costs for industry to reformulate their products if reformulation occurs. Government costs were estimated based on the FDA’s budget report and Nutrition Review Project (eAppendix 5 and eTables 2-4 in the Supplement),^15,16^ and industry costs were estimated based on the FDA’s regulatory impact analysis that accounted for variations in factors including product formula complexity, company size, reformulation type, and compliance period, providing a more accurate cost estimate than a standard per-product cost approach.^8^

Direct medical costs for cancer care were extracted from the Surveillance, Epidemiology, and End Results–Medicare linked database for 3 phases of cancer care: initial, continuing, and end-of-life (eAppendix 6, eTable 5, eTable 11, and eTable 12 in the Supplement).^14,17^ Indirect costs included productivity loss due to missed workdays or disability and patient time costs associated with receiving care, derived from published studies using the Medical Expenditure Panel Survey data.^18,19,20,21^

### Cost-effectiveness and Sensitivity Analyses

Following the recommendations from the Second Panel on Cost-effectiveness in Health and Medicine,^22^ we evaluated the policy association with health outcomes by projecting the number of new cancer cases and cancer deaths averted and quality-adjusted life-years (QALYs) gained and cost-effectiveness from health care and societal perspectives. The health care perspective assessed net costs as the government costs of implementing the policy minus savings in the direct medical costs for cancer care. The societal perspective assessed net costs as the difference between the total policy costs (including government and industry costs) and the health-related cost savings (including direct and indirect costs for cancer care). All costs were inflated to 2015 US dollars using the Consumer Price Index or Personal Health Care Index, with all costs and QALYs discounted at 3% annually. Incremental cost-effectiveness ratios (ICERs) were calculated as the difference in costs divided by the difference in QALYs between policy vs no policy. ICERs falling below a willingness-to-pay threshold of $150 000 per QALY gained were considered to be cost-effective.^23,24^ Cost-effectiveness analysis was further conducted among population subgroups by age, sex, and race/ethnicity to evaluate policy associations with health disparities.

We performed 1-way sensitivity analyses by varying key input parameters, including policy association, diet-BMI association, changes in medical expenditures associated with cancer care, policy implementation costs, and discounting rate. In addition, we conducted threshold analyses to identify the minimum policy change needed for achieving cost-saving or cost-effective status, evaluated the cost-effectiveness at a 10-year horizon for stakeholders interested in near-term returns, and further identified the time frame during which the policy reached cost-effectiveness. Probabilistic sensitivity analyses were conducted to incorporate the probability distributions around the point estimates of all parameters jointly (Table 1). A total of 1000 Monte Carlo simulations were performed, and 95% uncertainty intervals (UIs) were estimated based on the 2.5 and 97.5 percentiles of the 1000 simulations. All analyses were conducted using SAS, version 9.4 (SAS Institute Inc) and R, version 3.3.1 (R Foundation).

## Results

### Population Characteristics

The simulated cohort of US adults in 2015 had a mean age of 47.8 years, comprising 65.0% non-Hispanic White individuals and 71.4% overweight or obese adults (eTable 7 in the Supplement). The mean consumption of added sugars was 73.0 g/d from all foods (13.9% of daily calories) and 52.6 g/d from packaged foods and beverages (10.0% of daily calories). Higher amounts of added sugars were consumed by individuals aged 65 years or older (56.9 g/d), women (57.7 g/d), and non-Hispanic Black individuals (55.1 g/d) compared with other groups. Implementing the added-sugar labeling policy was associated with a 3.46-g/d decrease in added sugar intake, corresponding to reductions of BMI in healthy-weight (0.02 points) and overweight/obese (0.04 points) individuals.

### Health Association

Based on consumer behavior alone, implementing the FDA added-sugar labeling policy was associated with a decrease of 30 000 (95% UI, 21 600-39 300) new cancer cases (0.09% reduction of incident cancer) and 17 100 (95% UI, 12 400-22 700) cancer deaths (0.08% reduction of total cancer deaths), and an increase of 116 000 (95% UI, 83 800-153 000) QALYs among 235 million US adults over a median follow-up of 34.4 years (Table 2). With additional industry reformulation, the policy was associated with a reduction of 65 000 (95% UI, 51 400-79 800) new cancer cases (0.19% reduction) and 36 300 (95% UI, 28 700-44 900) cancer deaths (0.18% reduction), and a gain of 252 000 (95% UI, 199 000-312 000) QALYs. By cancer type, the greatest number of new cancer cases averted were endometrial, postmenopausal breast, and kidney cancers and the greatest number of cancer deaths decreased were liver, postmenopausal breast, and endometrial cancers.

**Table 2.** Estimated Health Gains and Costs of the US Food and Drug Administration’s Added-Sugar Labeling on Reducing Cancer Burdens in the US Over 12 Years and a Lifetime^a^

Parameter	Added-sugar labeling policy, median (95% UI)			
	12 Years		Lifetime	
	Consumer Behavior	Consumer behavior + industry response	Consumer behavior	Consumer behavior + industry response
New cancer cases averted, No.				
Endometrial	1530 (957 to 2280)	3270 (2360 to 4350)	6240 (3500 to 9500)	15 400 (10 900 to 20 300)
Breast (postmenopausal)	1530 (892 to 2360)	3290 (2230 to 4630)	5760 (3360 to 8880)	13 900 (9980 to 19 000)
Kidney	935 (636 to 1300)	2030 (1580 to 2540)	5480 (4160 to 6940)	10 900 (8690 to 13 200)
Liver	616 (416 to 899)	1370 (1020 to 1800)	5080 (3920 to 6720)	9960 (7910 to 12600)
Esophageal adenocarcinoma	254 (149 to 398)	543 (366 to 759)	1580 (1140 to 2140)	3020 (2240 to 3910)
Pancreatic	294 (197 to 415)	626 (464 to 817)	1460 (1040 to 1920)	3100 (2420 to 3840)
Colorectal	308 (216 to 424)	675 (518 to 860)	1200 (897 to 1520)	2390 (1870 to 2950)
Stomach (cardia)	107 (61.4 to 172)	230 (150 to 336)	679 (473 to 1010)	1330 (942 to 1870)
Multiple myeloma	129 (86.8 to 193)	278 (197 to 386)	649 (436 to 932)	1400 (1000 to 1870)
Thyroid	171 (118 to 248)	363 (270 to 494)	611 (397 to 924)	1340 (944 to 1890)
Advanced prostate	79 (44 to 138)	175 (114 to 261)	380 (270 to 523)	702 (509 to 957)
Gallbladder	55 (39 to 76)	118 (92 to 153)	313 (233 to 406)	674 (544 to 836)
Ovarian	64 (37 to 103)	135 (87 to 204)	158 (65 to 291)	397 (198 to 636)
Total	6170 (4280 to 8460)	13 300 (10 400 to 17 000)	30 000 (21 600 to 39 300)	65 000 (51 400 to 79 800)
Cancer deaths prevented, No.				
Liver	350 (237 to 509)	766 (568 to 1010)	4440 (3420 to 5890)	8690 (6880 to 11 100)
Breast (postmenopausal)	183 (124 to 254)	374 (283 to 492)	3170 (1550 to 5570)	7910 (4950 to 11 800)
Endometrial	162 (97.1 to 242)	359 (255 to 477)	2100 (1230 to 3280)	5230 (3760 to 6990)
Kidney	149 (98.9 to 214)	323 (239 to 417)	2080 (1570 to 2670)	4170 (3360 to 5070)
Esophageal adenocarcinoma	135 (78.2 to 214)	289 (194 to 404)	1360 (987 to 1840)	2580 (1910 to 3330)
Pancreatic	185 (121 to 267)	395 (288 to 522)	1270 (903 to 1670)	2700 (2120 to 3350)
Colorectal	76 (53 to 105)	165 (126 to 212)	785 (592 to 1000)	1560 (1220 to 1940)
Stomach (cardia)	54 (30 to 86)	116 (74 to 170)	558 (392 to 834)	1090 (768 to 1540)
Multiple myeloma	39 (24 to 61)	84 (59 to 122)	389 (266 to 555)	835 (618 to 1100)
Gallbladder	31 (21 to 43)	66 (51 to 86)	263 (193 to 343)	572 (459 to 707)
Advanced prostate	12 (7 to 21)	27 (17 to 42)	170 (119 to 246)	314 (223 to 439)
Ovarian	22 (11 to 39)	48 (28 to 76)	114 (51 to 204)	285 (154 to 430)
Thyroid	2 (1 to 3)	5 (4 to 7)	25 (16 to 36)	53 (36 to 73)
Total	1430 (980 to 1940)	3080 (2400 to 3840)	17 100 (12 400 to 22 700)	36 300 (28 700 to 44 900)
Life years gained	2250 (1540 to 3100)	4830 (3750 to 6060)	78 300 (56 300 to 105 600)	168 117 (132 000 to 209 000)
QALYs gained	9640 (6740 to 13 600)	20 800 (16 000 to 27 200)	116 000 (83 800 to 153 000)	252 000 (199 000 to 312 000)
Changes in health-related costs, cancer only, millions, $^b^^,^^c^				
Medical	−364 (−490 to −253)	−779 (−971 to −613)	−1600 (−2030 to −1190)	−3400 (−4080 to −2720)
Patient time	−18.0 (−25.3 to −12.2)	−38.1 (−49.6 to −29.0)	−114 (−151 to −80.7)	−252 (−311 to −200)
Productivity	−111 (−150 to −75.9)	−234 (−297 to −183)	−669 (−890 to −478)	−1480 (−1820 to −1170)
Policy implementation costs, millions, $^b^^,^^c^				
Government	6.89 (5.96 to 8.30)	6.88 (5.91 to 8.52)	9.24 (7.18 to 12.8)	9.30 (7.28 to 12.5)
Administration	4.53 (4.29 to 4.76)	4.53 (4.31 to 4.79)	4.53 (4.29 to 4.80)	4.53 (4.29 to 4.77)
Monitoring	2.36 (1.41 to 3.74)	2.34 (1.41 to 3.97)	4.69 (2.75 to 8.29)	4.77 (2.69 to 7.96)
Industry	1660 (1410 to 1960)	2090 (1820 to 2410)	1660 (1410 to 1960)	2540 (2240 to 2880)
Compliance	1660 (1410 to 1960)	1660 (1400 to 1960)	1660 (1410 to 1960)	1660 (1400 to 1970)
Reformulation^d^	NA	427 (349 to 516)	NA	869 (718 to 1061)
Net costs, cancer only, millions, $^b,c,e^				
Societal perspective	1170 (871 to 1520)	1050 (675 to 1410)	−704 (−1450 to −44.5)	−2570 (−3730 to −1450)
Health care perspective	−357 (−483 to −247)	−773 (−964 to −607)	−1590 (−2020 to −1180)	−3390 (−4070 to −2710)
ICER ($/QALY)^f^				
Societal perspective	122 000 (70 600 to 207 000)	51 400 (26 800 to 84 700)	Cost to saving	Cost to saving
Health care perspective	Cost to saving	Cost to saving	Cost to saving	Cost to saving

Abbreviations: ICER, incremental cost-effectiveness ratio; UI, uncertainty interval; QALY, quality-adjusted life year.

^a^ Values are the median estimates (95% uncertainty intervals) of each distribution of 1000 simulations.

^b^ Health-related costs were inflated to 2015 US dollars using the Personal Health Care (PHC) index. Policy intervention costs were inflated to 2015 US dollars using the Consumer Price Index. Negative costs represent savings.

^c^ Costs are medians from 1000 simulations so may not add up to totals.

^d^ In the scenario considering policy association with consumer behavior alone, there is no policy cost for industry reformulation.

^e^ Net costs were calculated as policy costs minus health-related costs from reduced cancer burdens. Societal perspective includes health care cost, patient time costs, productivity costs, and policy implementation costs; government perspective included policy costs relevant to policy implementation and program monitoring and evaluation and medical costs.

^f^ ICER threshold was evaluated at $150 000/QALY.

### Economic Impact

**Figure 1.**
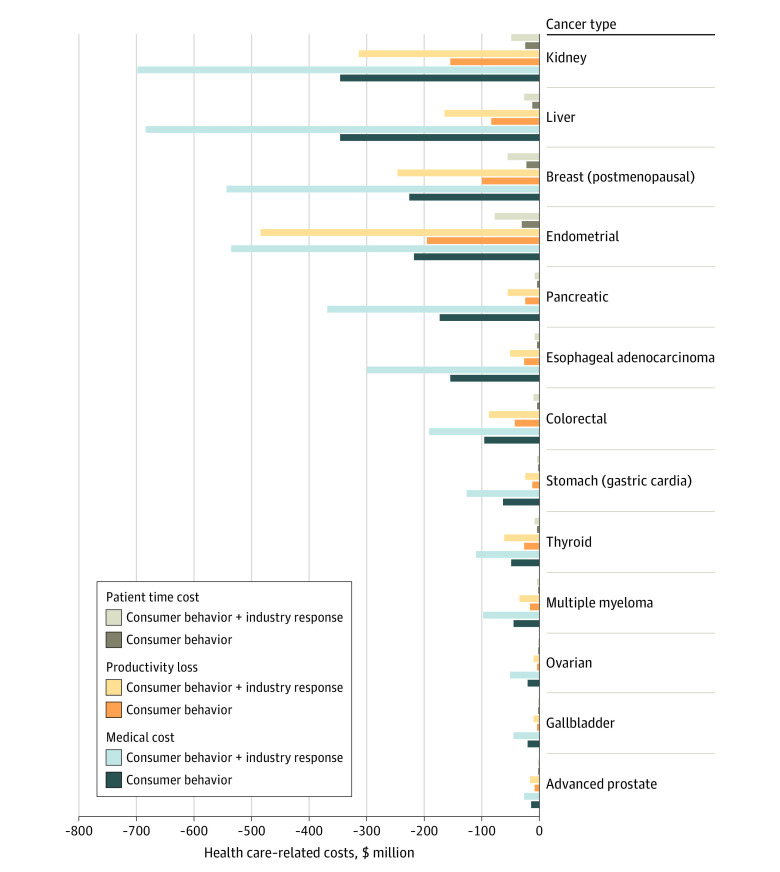
Estimated Health Care–Related Cost Savings Associated With Added-Sugar Labeling by Cancer Type Over a Lifetime

Implementing the policy would cost the government an estimated $9.24 million ($7.18 million-$12.8 million) and the industry $1660 million ($1410 million-$1960 million) in compliance costs over a lifetime of the current US population (Table 2). The policy was associated with savings of $1600 (95% UI, $1190 million-$2030 million) direct medical costs, $669 million (95% UI, $478 million-$890 million) productivity loss costs, and $114 million (95% UI, $80.7 million-$151 million) patient time costs for cancer care. With industry reformulation, the policy would cost the industry an additional $869 million ($718 million-$1060 million) and was associated with greater health care cost savings, including $3400 (95% UI, $2720-$4080) in direct medical costs, $1480 (95% UI, $1170-$1820) in direct productivity loss costs, and $252 (95% UI, $200-$311) million in direct and patient time costs. The top 3 cancers with the highest medical cost savings were kidney, liver, and postmenopausal breast cancers (Figure 1 and eTable 13 in the Supplement).

The policy intervention was cost-saving and associated with savings of $704 million (95% UI, $45 million-$1450 million) from a societal perspective and $1590 million (95% UI, $1180 million-$2020 million) a health care perspective (Table 2). Further industry reformulation would increase the saving to $2570 million (95% UI, $1450 million-$3730 million) from the societal perspective and $3390 million (95% UI, $2710 million-$4070 million) from the health care perspective.

### Policy Associations Among Subgroups

The policy was associated with greater health gains per 100 000 individuals among young adults (age, 20-44 years; 15 new cancer cases averted) than middle-aged adults (age, 45-54 years; 9 new cancer cases averted), women than men (14 vs 12 new cancer cases averted), and non-Hispanic Black individuals (15 new cancer cases averted) than Hispanic individuals (10 new cancer cases averted) (Table 3). The number of cancer deaths averted, life-years and QALYs gained, health-related costs saved, and net costs followed the same pattern. For example, the policy was associated with more cancer deaths prevented among young adults (age, 20-44 years; 10 deaths per 100 000 individuals) than older adults (age, ≥65 years; 4 deaths per 100 000 individuals), and non-Hispanic Black individuals (9 deaths per 100 000 individuals) than Hispanic individuals (6 deaths per 100 000 individuals). Industry reformulation doubled the outcomes across age, sex, and race/ethnicity groups, with similar disparity patterns observed, such as 32 new cancer cases averted per 100 000 young adults vs 28 cases per 100 000 middle-aged adults, and 21 cancer deaths prevented per 100 000 non-Hispanic Black individuals vs 16 deaths per 100 000 Hispanic individuals (eFigures 2-5 in the Supplement). Findings were also broadly similar within each cancer type (eTable 14 and eTable 15 in the Supplement).

**Table 3.** Estimated New Cancer Cases and Deaths Prevented by FDA’s Added-Sugar Labeling Policy Among US Adults Over a Lifetime by Age, Sex, and Race/Ethnicity^a^

Parameter	Consumer behavior		Consumer behavior + industry response	
	Total, No. (95% UI)	No. per 100 000 (95% UI)	Total, No. (95% UI)	No. per 100 000 (95% UI)
**No. of new cancer cases averted**				
Age, y				
20-44	15 800 (9840-23 400)	15.0 (9.39-22.3)	33 000 (23 200-44 800)	31.5 (22.2-42.8)
45-54	3910 (733-8120)	9.19 (1.72-19.1)	11 200 (6220-17 500)	26.3 (14.6-41.1)
55-64	5320 (2780-8980)	13.2 (6.88-22.2)	11 100 (6680-16 500)	27.5 (16.5-40.9)
≥65	4520 (2520-7720)	9.53 (5.31-16.3)	9230 (5420-14100)	19.5 (11.4-29.7)
Sex				
Female	16 400 (9660-25 000)	13.6 (8.00-20.7)	40 600 (29 200-53 300)	33.6 (24.2-44.2)
Male	13 200 (9560-17 900)	11.6 (8.35-15.7)	24 400 (18 300-31 300)	21.3 (16.0-27.4)
Race/ethnicity				
Non-Hispanic White	20 100 (13 200-29 000)	12.9 (8.51-18.7)	42 900 (31 300-56 800)	27.5 (20.1-36.5)
Non-Hispanic Black	4200 (1820-7810)	14.7 (6.38-27.4)	9470 (5910-14 100)	33.2 (20.7-49.6)
Hispanic	3540 (1380-6170)	9.86 (3.85-17.2)	9310 (5390-13 800)	25.9 (15.0-38.4)
Other	1580 (881-2470)	10.5 (5.84-16.4)	2960 (2000-4060)	19.7 (13.2-26.9)
**No. of cancer deaths prevented**				
Age, y				
20-44	10 100 (6340-15 000)	9.67 (6.05-14.3)	21 100 (14 800-28 300)	20.1 (14.1-27.0)
45-54	2220 (696-4080)	5.22 (1.64-9.61)	5870 (3410-8530)	13.8 (8.03-20.1)
55-64	2560 (1440-4050)	6.33 (3.57-10.0)	5180 (3430-7450)	12.8 (8.48-18.4)
≥65	1970 (1130-3170)	4.16 (2.39-6.68)	4040 (2460-5800)	8.51 (5.19-12.2)
Sex				
Female	7990 (4550-12400)	6.62 (3.77-10.3)	19 800 (13 800-26 800)	16.4 (11.4-22.2)
Male	8840 (6450-12100)	7.73 (5.63-10.6)	16 400 (12 200-21 100)	14.3 (10.7-18.5)
Race/ethnicity				
Non-Hispanic White	10 900 (7320-15 500)	7.02 (4.70-9.95)	22 600 (16500-30 100)	14.5 (10.6-19.3)
Non-Hispanic Black	2700 (1070-5150)	9.48 (3.76-18.1)	6110 (3670-9290)	21.4 (12.9-32.6)
Hispanic	2270 (984-3880)	6.33 (2.74-10.8)	5730 (3310-8450)	16.0 (9.21-23.5)
Other	891 (504-1410)	5.91 (3.34-9.33)	1680 (1110-2340)	11.2 (7.35-15.5)

Abbreviation: UI, uncertainty interval.

^a^ Values are the median estimates (95% UI) of each distribution of 1000 simulations.

### Sensitivity Analyses

The cost-effectiveness of the policy was most sensitive to varying assumptions of policy impact, diet-BMI association, and discounting rate (eTable 17 and eFigure 6 in the Supplement). From the societal perspective, the minimal policy impact needed for consumer behavior for the policy to be cost-saving was 4.55%; with additional industry reformulation, the minimal policy impact for reformulation of consumer behavior was 3.23% and, for industry reformulation, 4.04% for the policy to be cost-saving (eTable 18 in the Supplement). When a different time horizon was used, the associated health gains were smaller over 10 to 12 years compared with those over a lifetime; however, the policy was still cost-saving from the health care perspective (Table 2 and eTable 16 in the Supplement).

**Figure 2.**
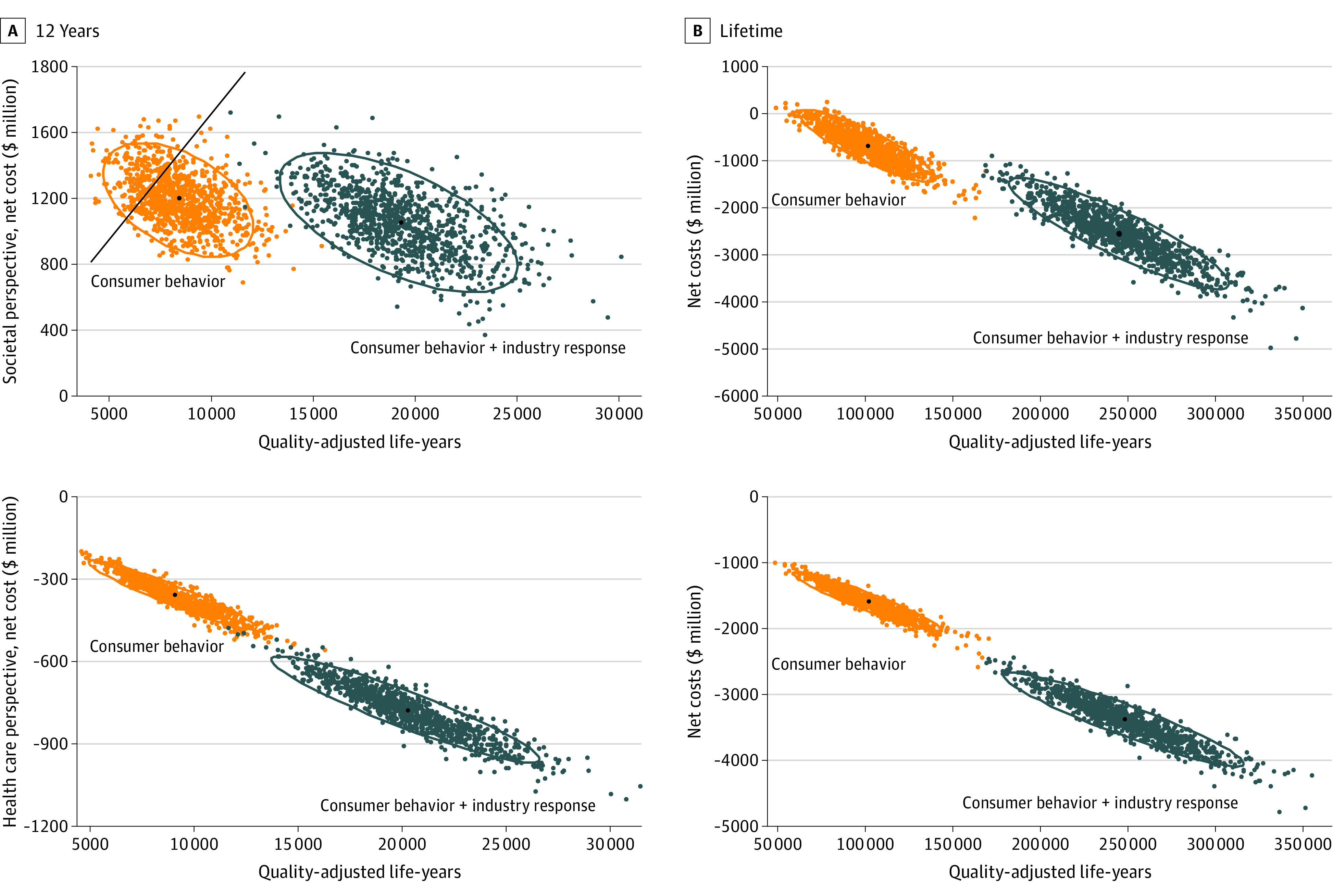
Probabilistic Sensitivity Analyses for Cost-effectiveness of Added-Sugar Labeling Over 12 Years and a Lifetime

From the societal perspective, the policy, based on consumer responses alone, would achieve cost-saving for nearly 100% of the 1000 lifetime simulations because nearly all results decreased to below zero (Figure 2). At 12 years, the policy would have a 78% probability of having an ICER less than $150 000/QALY (78% of simulated results decreased below the threshold line). When adding industry reformulation, this probability would increase to 100% over a lifetime and by 12 years. From the health care perspective, the policy would be cost-saving in 1000 simulations, based on either consumer responses alone or additional industry reformulations over a lifetime and when limiting the analytic time horizon to 12 years for both scenarios.

## Discussion

Using a probabilistic simulation model, our study estimated that implementing the added-sugar labeling policy was associated with a 3.46-g/d decrease in added sugar intake, corresponding to reductions of BMI in healthy-weight (0.02 points) and overweight/obese (0.04 points) individuals. The policy was estimated to be associated with approximately 30 000 new cancer cases and 17 100 cancer deaths among US adults over a lifetime and with substantial health care savings, resulting in net savings of $704 million from the societal perspective and $1590 million from the health care perspective. With additional industry reformulation, the policy was associated with approximately doubled health gains and economic benefits. Greater health gains and cost savings were expected among young adults, women, and non-Hispanic Black individuals compared with other population subgroups.

Our findings suggest that the added-sugar labeling policy could be a cost-saving strategy for cancer prevention. Earlier simulation studies reported that medical screenings were cost-effective interventions in the primary or secondary prevention of specific cancers.^30,31,32^ For example, mammography screening starting at age 45 years was estimated to have an ICER of $40 135/QALY among a large cohort of women born in the 1960s over a lifetime.^30^ Colonoscopy screening starting at age 45 years achieved an ICER of $33 900/QALY among US men and women,^31^ and prostate-specific antigen screening had an ICER of $70 831 to $136 332/QALY among a cohort of US men beginning at age 40 years over a lifetime.^32^ These cancer screening strategies are cost-effective, but none of them achieved net savings. Our results suggest that a population-based nutrition intervention, such as the added-sugar labeling policy, is not only cost-effective but also cost-saving. These findings underscore the need to consider and prioritize nutrition-related policy interventions as cost-effective or cost-saving strategies for cancer prevention.

Modest industry responses to the labeling policy could be associated with substantial additional health gains and economic benefits for obesity-related cancers. Earlier studies reported that, after the US Congress passed a national law requiring chain restaurants with 20 or more outlets to list calories on menus in 2010, the mean calorie content per menu item fell from 327 kcal in 2008 to 318 kcal in 2015 in 44 of the 100 largest chain restaurants.^33^ A meta-analysis of interventional studies found that package, menu, and other point-of-purchase labeling policies were associated with industry reformulations, including an 8.9% reduction in sodium content.^7^ Thus, with the passage of the added-sugar labeling, government and advocacy strategies should be prioritized to encourage industry reformulations for achieving larger benefits.

### Limitations

The study had limitations. First, our investigation cannot prove a causal relationship between the implementation of this policy and cancer outcomes. Yet, cost-effectiveness analysis provides flexibility to test and evaluate different policy scenarios and reasoned assumptions. Second, we did not include potential health benefits on other sugar- or obesity-associated diseases (eg, diabetes, cardiovascular diseases) or the potential associations with reducing cancer burdens through reduced childhood obesity. Thus, the health benefits and cost savings of the policy could be underestimated. Third, in the absence of direct evidence, we used the estimated association of menu calorie labeling and calorie reduction as a proxy for added-sugar labeling, and limited evidence has suggested an association between menu calorie labeling or nutrition fact information and eating behaviors. However, in the sensitivity analyses with more conservative estimates, the policy remained cost-effective from both health care and societal perspectives. Fourth, insufficient data were available to incorporate potentially differing responses to labeling policy by socioeconomic status. Some studies,^34,35,36^ but not others,^7,37^ suggest that individuals with lower educational levels or income may respond less to nutrition labeling than those with greater educational levels or income. However, such a varying response might alter the relative benefits within the subpopulation but would be unlikely to alter the robust overall cost-savings.

## Conclusions

This economic evaluation is, to our knowledge, among the first to suggest that the added-sugar labeling policy is cost-saving and associated with reduced costs and lower cancer rates. Nutrition policies can play important roles in reducing cancer burdens and disparities in the US.

## Notes

Supplement. **eAppendix 1.** Baseline Cancer Incidence and Methods of Cancer Incidence Projections for 13 Types of Cancer **eAppendix 2.** Cancer Survival for 13 Types of Cancer **eTable 1.** Period Method for Five-Year Relative Survival for 2014 **eAppendix 3.** Methods of Estimating the Health-Related Quality of Life Among 13 Types of Cancer **eAppendix 4.** Estimate the Association Between Added Sugar Labeling Policy and Added Sugar Intake **eAppendix 5.** Methods of Estimating Policy Implementation Costs **eTable 2.** Implementation Cost Estimates for the Nutrition Facts Added Sugar Labeling Policy (in 2015 US Dollars) **eTable 3.** Discounting Factor in Each Year Over a Lifetime **eTable 4.** Population Size of People Who are Alive in Each Year Over a Lifetime (in Millions) **eAppendix 6.** Annual Health-Related Costs Among Cancer Patients and the General Population Without Cancer **eTable 5.** Description of Data Source of Health-Related Expenditures **eTable 6.** Defining Population and 32 Subgroups **eTable 7.** Characteristics of US Adults Aged 20 Years or Older Participated in the NHANES, 2013-2016 **eTable 8.** Consumption of Added Sugar From Foods and Beverages Among US Adults Participated in 2013-2016 NHANES, by 32 Subgroups **eTable 9.** Relative Risk Estimates of Etiologic Relationships Between Body Mass Index (BMI) and Cancer **eTable 10.** Health-Related Quality of Life Among US Cancer Patients Aged 20 Years or Older, by Cancer Type and Phase of Care **eTable 11.** Baseline Medical Costs, Productivity Loss, and Patient Time Costs Among US Cancer Patients Aged 20 Years or Older, by Cancer Type **eTable 12.** Baseline Medical Costs, Productivity Loss, and Patient Time Costs Among the General Population Aged 20 Years or Older in the US, by 32 Subgroups **eTable 13.** Estimated Changes in Health-Related Costs Associated With Nutrition Facts Added Sugar Labeling on Reducing Cancer Burdens in the US Over a Lifetime, by Cancer Type **eTable 14.** Estimated New Cancer Cases Averted by Nutrition Facts Added Sugar Labeling Policy in the US by Age, Gender, Race/Ethnicity, and Cancer Type Over a Lifetime **eTable 15.** Estimated Cancer Deaths Reduced by Nutrition Facts Added Sugar Labeling Policy in the US by Age, Gender, Race/Ethnicity, and Cancer Type Over a Lifetime **eTable 16.** Estimated Health Gains and Costs Associated With Nutrition Facts Added Sugar Labeling on Reducing Cancer Burdens in the US Over 10 Years **eTable 17**. Estimated Health Gains and Costs Associated With Nutrition Facts Added Sugar Labeling on Reducing Cancer Burdens in the US Over a Lifetime, One-Way Sensitivity Analyses at 50% of the Policy Impact and the Diet-BMI Association **eTable 18.** Estimated Health Gains and Costs Associated With Nutrition Facts Added Sugar Labeling on Reducing Cancer Burdens in the US Over a Lifetime, Threshold Analyses on Policy Impact **eFigure 1.** Diet and Cancer Outcome Model (DiCOM) **eFigure 2.** Estimated Reduced New Cancer Cases and Deaths Associated With Nutrition Facts added Sugar Labeling Policy in the US by Age, Gender, Race/Ethnicity, and Cancer Type Over a Lifetime **eFigure 3.** Estimated Life Years and QALYs Gained Associated With Nutrition Facts Added Sugar Labeling Policy in the US by Age, Gender, and Race/Ethnicity Over a Lifetime **eFigure 4.** Estimated Changes of Health-Related Costs Associated With Nutrition Facts Added Sugar Labeling Policy in the US by Age, Gender, Race/Ethnicity and Cancer Type Over a Lifetime **eFigure 5.** Estimated Net Costs From Societal and Healthcare Perspectives Associated With Nutrition Facts Added Sugar Labeling Policy in the US by Age, Gender, and Race/Ethnicity Over a Lifetime **eFigure 6.** One-Way Sensitivity Analysis of Net Costs of Nutrition Facts Added Sugar Label and Obesity-Associated Cancer Rates to Varying Assumptions of Key Input Parameters From (A) Societal Perspective and (B) Healthcare Perspective **eReferences**
