# Supplementary material for: Cost-effectiveness Analysis of Nutrition Facts Added-Sugar Labeling and Obesity-Associated Cancer Rates in the US
Source: JAMA Netw Open. 2021 Apr 27;4(4):e217501. doi: 10.1001/jamanetworkopen.2021.7501 (PMC8080223; doi:10.1001/jamanetworkopen.2021.7501)
Supplement: Supplement. — eAppendix 1. Baseline Cancer Incidence and Methods of Cancer Incidence Projections for 13 Types of Cancer eAppendix 2. Cancer Survival for 13 Types of Cancer eTable 1. Period Method for Five-Year Relative Survival for 2014 eAppendix 3. Methods of Estimating the Health-Related Quality of Life Among 13 Types of Cancer eAppendix 4. Estimate the Association Between Added Sugar Labeling Policy and Added Sugar Intake eAppendix 5. Methods of Estimating Policy Implementation Costs eTable 2. Implementation Cost Estimates for the Nutrition Facts Added Sugar Labeling Policy (in 2015 US Dollars) eTable 3. Discounting Factor in Each Year Over a Lifetime eTable 4. Population Size of People Who are Alive in Each Year Over a Lifetime (in Millions) eAppendix 6. Annual Health-Related Costs Among Cancer Patients and the General Population Without Cancer eTable 5. Description of Data Source of Health-Related Expenditures eTable 6. Defining Population and 32 Subgroups eTable 7. Characteristics of US Adults Aged 20 Years or Older Participated in the NHANES, 2013-2016 eTable 8. Consumption of Added Sugar From Foods and Beverages Among US Adults Participated in 2013-2016 NHANES, by 32 Subgroups eTable 9. Relative Risk Estimates of Etiologic Relationships Between Body Mass Index (BMI) and Cancer eTable 10. Health-Related Quality of Life Among US Cancer Patients Aged 20 Years or Older, by Cancer Type and Phase of Care eTable 11. Baseline Medical Costs, Productivity Loss, and Patient Time Costs Among US Cancer Patients Aged 20 Years or Older, by Cancer Type eTable 12. Baseline Medical Costs, Productivity Loss, and Patient Time Costs Among the General Population Aged 20 Years or Older in the US, by 32 Subgroups eTable 13. Estimated Changes in Health-Related Costs Associated With Nutrition Facts Added Sugar Labeling on Reducing Cancer Burdens in the US Over a Lifetime, by Cancer Type eTable 14. Estimated New Cancer Cases Averted by Nutrition Facts Added Sugar Labeling Policy in the US by Age, G [file jamanetwopen-e217501-s001.pdf]

Supplementary Online Content

Du M, Griecci CF, Cudhea FF, et al; Food-PRICE Project. Cost-effectiveness analysis of nutrition facts added-sugar labeling and obesity-associated cancer rates in the US. *JAMA Netw Open*. 2021;4(4):e217501. doi:10.1001/jamanetworkopen.2021.7501

eAppendix 1. Baseline Cancer Incidence and Methods of Cancer Incidence Projections for 13 Types of Cancer
eAppendix 2. Cancer Survival for 13 Types of Cancer
eTable 1. Period Method for Five-Year Relative Survival for 2014
eAppendix 3. Methods of Estimating the Health-Related Quality of Life Among 13 Types of Cancer
eAppendix 4. Estimate the Association Between Added Sugar Labeling Policy and Added Sugar Intake
eAppendix 5. Methods of Estimating Policy Implementation Costs
eTable 2. Implementation Cost Estimates for the Nutrition Facts Added Sugar Labeling Policy (in 2015 US Dollars)
eTable 3. Discounting Factor in Each Year Over a Lifetime
eTable 4. Population Size of People Who are Alive in Each Year Over a Lifetime (in Millions)
eAppendix 6. Annual Health-Related Costs Among Cancer Patients and the General Population Without Cancer
eTable 5. Description of Data Source of Health-Related Expenditures
eTable 6. Defining Population and 32 Subgroups
eTable 7. Characteristics of US Adults Aged 20 Years or Older Participated in the NHANES, 2013-2016
eTable 8. Consumption of Added Sugar From Foods and Beverages Among US Adults Participated in 2013-2016 NHANES, by 32 Subgroups
eTable 9. Relative Risk Estimates of Etiologic Relationships Between Body Mass Index (BMI) and Cancer
eTable 10. Health-Related Quality of Life Among US Cancer Patients Aged 20 Years or Older, by Cancer Type and Phase of Care
eTable 11. Baseline Medical Costs, Productivity Loss, and Patient Time Costs Among US Cancer Patients Aged 20 Years or Older, by Cancer Type
eTable 12. Baseline Medical Costs, Productivity Loss, and Patient Time Costs Among the General Population Aged 20 Years or Older in the US, by 32 Subgroups
eTable 13. Estimated Changes in Health-Related Costs Associated With Nutrition Facts Added Sugar Labeling on Reducing Cancer Burdens in the US Over a Lifetime, by Cancer Type
eTable 14. Estimated New Cancer Cases Averted by Nutrition Facts Added Sugar Labeling Policy in the US by Age, Gender, Race/Ethnicity, and Cancer Type Over a Lifetime
eTable 15. Estimated Cancer Deaths Reduced by Nutrition Facts Added Sugar Labeling Policy in the US by Age, Gender, Race/Ethnicity, and Cancer Type Over a Lifetime
eTable 16. Estimated Health Gains and Costs Associated With Nutrition Facts Added Sugar Labeling on Reducing Cancer Burdens in the US Over 10 Years
eTable 17. Estimated Health Gains and Costs Associated With Nutrition Facts Added Sugar Labeling on Reducing Cancer Burdens in the US Over a Lifetime, One-Way Sensitivity Analyses at 50% of the Policy Impact and the Diet-BMI Association
eTable 18. Estimated Health Gains and Costs Associated With Nutrition Facts Added Sugar Labeling on Reducing Cancer Burdens in the US Over a Lifetime, Threshold Analyses on Policy Impact
eFigure 1. Diet and Cancer Outcome Model (DiCOM)
eFigure 2. Estimated Reduced New Cancer Cases and Deaths Associated With Nutrition Facts added Sugar Labeling Policy in the US by Age, Gender, Race/Ethnicity, and Cancer Type Over a Lifetime
eFigure 3. Estimated Life Years and QALYs Gained Associated With Nutrition Facts Added Sugar Labeling Policy in the US by Age, Gender, and Race/Ethnicity Over a Lifetime
eFigure 4. Estimated Changes of Health-Related Costs Associated With Nutrition Facts Added Sugar Labeling Policy in the US by Age, Gender, Race/Ethnicity and Cancer Type Over a Lifetime
eFigure 5. Estimated Net Costs From Societal and Healthcare Perspectives Associated With Nutrition Facts Added Sugar Labeling Policy in the US by Age, Gender, and Race/Ethnicity Over a Lifetime
eFigure 6. One-Way Sensitivity Analysis of Net Costs of Nutrition Facts Added Sugar Label and Obesity-Associated Cancer Rates to Varying Assumptions of Key Input Parameters From (A) Societal Perspective and (B) Healthcare Perspective
eReferences

This supplementary material has been provided by the authors to give readers additional information about their work.

© 2021 Du M et al. *JAMA Network Open.*

eAppendix 1. Baseline cancer incidence and methods of cancer incidence projections for 13 types of cancer

We estimated the cancer incidence rate projections for the defined 32 demographic subgroups as inputs for the DiCOM model. We obtained age adjusted incidence rates, then projected rates based on historical trends, and then applied a cohort period age shifting method to calculate incidence rates for each of 32 subgroups over their lifetime.

We first obtained age-adjusted incidence rates for each year (2006-2015) from the United States Cancer Statistics which combines data from the Surveillance, Epidemiology, and End Results (SEER) database and the Centers for Disease Control and Prevention's National Program of Cancer Registries (NPCR) database.[1] An age-adjusted rate is a weighted average of the age-specific rates, where the weights are the proportions of persons in the corresponding age groups of a standard population. The potential confounding effect of age is reduced when comparing age-adjusted rates computed using the same standard population.

We then projected age-adjusted cancer incidence based on historical trends. We used the incidence data estimated from the most recent 10 years, 2006-2015, to predict the future 15 years, 2016-2030 and then held the incidence rate constant for all subsequent years.[2] To do so, we estimated the average annual percent change (AAPC) using age-adjusted incidence rates from 2006 to 2015, then applied it to the 2015 baseline incidence to project future incidence for each cancer type by subgroup.[3] The estimated annual percent change was calculated for each cancer site and 32 subgroups by fitting a regression line to the natural logarithm of the age-adjusted rates (I) in years 2006 through 2015 (y). The equation for AAPC: $\ln(I) = \alpha + \beta y$, where α and β were coefficients to be estimated and y is calendar year.[2,3] The average annual percent change (AAPC) is a summary measure of the trend over a pre-specified fixed interval. It allows us to use a single number to describe the average APCs over a period of multiple years. It is valid even if the model indicates that there were changes in trends during those years. It is computed as a weighted average of the APCs, with the weights equal to the length of the APC interval.

We combined the AAPC projected incidence rates with the projected US population data and apply the cohort-period method to estimate the "crude" projected cancer incidence in each of the 32 subgroups from 2016 and 2095 incidence rates that will be used in the DiCOM model. US population estimates by single year of age, race/ethnicity, and sex will be extracted from the National Interim Projections of the US population via the US Census Bureau website. The projections series use the official estimates of the resident population on July 1, 2016 as the base for projecting the U.S. population from 2017 to 2060.[4] The series use the cohort-component method and historical trends in births, deaths, and international migration to project the future size and composition of the national population.

We have made two assumptions about this incidence rate. The population projection data are through year 2060 so we will assume incidence rates to be constant from year 2060 onward. We also assumed the population dies once they hit 100 years old and the model will then apply an incidence rate of 0 for any remaining years through year 2095. The DiCOM model will take into account the second assumption that death occurs at 100 years old.

© 2021 Du M et al. *JAMA Network Open.*

eAppendix 2. Cancer survival for 13 types of cancer

We estimated the 5-year relative survival for the defined 32 demographic subgroups. We obtained five-year relative survival rates using the period analysis method from the United States Cancer Statistics which incorporates data from the Surveillance, Epidemiology, and End Results (SEER) database.[1] The five-year survival for 2014, which was the most recent available data at time of analysis, was used. These rates were extracted for each cancer type and by the defined 32 demographic subgroups for each cancer type. The rates will be on a scale 0-1.

Relative survival is a net survival measure representing cancer survival in the absence of other causes of death. Relative survival is defined as the ratio of the proportion of observed survivors in a cohort of cancer patients to the proportion of expected survivors in a comparable set of cancer free individuals.[5] Relative survival is the preferred method to estimate survival from cancer registry data.

Period analysis is a method which enhances up-to-date monitoring of survival.[6,7] In contrast to traditional cohort analysis of survival, period analysis derives long-term survival estimates exclusively from the survival experience of patients within some recent calendar period.[6,7] Three-year intervals were chosen which results in years 2008-2014 used to calculate 5-year survival. Using seven years of data to calculate 5-year survival is standard method used by SEER and used in SEER publications.[8]

The first interval will contribute to the one year survival and will use cases diagnosed in 2012-2014, the second interval will contribute to the two year survival and will use cases diagnosed in 2011-2013, the third interval will contribute to the three year survival and will use cases diagnosed in 2010-2012, the fourth interval will contribute to the four year survival and will use cases diagnosed in 2009-2011 and the fifth interval will contribute to the five year survival and will use cases diagnosed in 2008-2010.

This analysis therefore used 2008-2014 diagnoses to calculate for 5-year relative survival for 2014. The highlighted orange boxes represent survival contributions for each year of diagnosis and year of follow-up (e**Table 1**). The annual probability of death was calculated as 1-exp[ln(5-year relative survival)/5].

© 2021 Du M et al. *JAMA Network Open.*

eTable 1. Period method for five-year relative survival for 2014

	YEARS OF DIAGNOSIS														
	2000	2001	2002	2003	2004	2005	2006	2007	2008	2009	2010	2011	2012	2013	2014
1															
2															
3															
4															
5															

© 2021 Du M et al. *JAMA Network Open.*

eAppendix 3. Methods of estimating health related quality of life among 13 types of cancer

Health utility values range from 0 (dead) to 1 (perfect health and were assigned for each cancer type and by phase of care (initial, continuous, end of life), if available. We first searched databases for systematic reviews pertaining to utility weights or HRQOL measures for each cancer type of interest separately. We started with PubMed and searched Google Scholar if needed. The following search string was used for each cancer type: ("health related quality of life" OR "HRQOL" OR "quality of life" OR "QOL" OR "preference weight*" OR “utility weight*” OR “health state utilit*” OR “health utility*”) AND (“cancer of interest”) AND ("cancer" OR “neoplasm*”) AND ("review" OR “systematic review”).

When an appropriate systematic review was identified, we read the articles included in the review and determined if the paper met the following data needs. Data Extraction Hierarchy: 1) cancer type specific to the type of interest; 2) consistent in instrument used, prefer EQ-5D whenever available; 3) US samples preferred; 4) phase of care (assume same utility weights by phase if phase of care data were not available). If no systematic reviews available, we searched for individual studies about the utility weights of the cancer of interest. Additionally, check how often the paper is cited to see if it is a frequently used utility weight.

© 2021 Du M et al. *JAMA Network Open.*

eAppendix 4. Estimate the association between added sugar labeling policy and added sugar intake

In order to understand the impact of the Nutrition Facts added sugar labeling policy, we must understand how both consumers and industry will respond to the policy as illustrated by the logical framework.

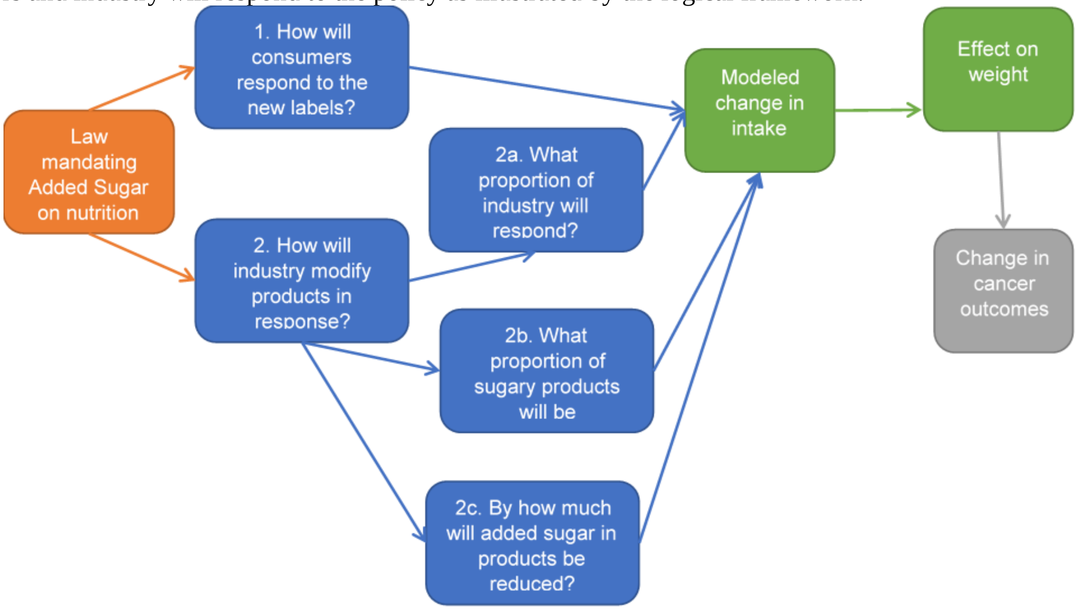

Because there is no much literature on the impact of added sugar labeling specifically, some assumptions must be made using the best available evidence from a mixture of sources. In Huang et al., the authors used the association estimate of calorie labeling, a reduction of intake by 6.6% (95% CI: 4.4% to 8.8%), as a proxy for added sugar labeling impact.[9,10] This policy impact estimate was chosen to represent a more conservative estimate than the larger impact observed from labeling on other dietary constituents such as sodium and *trans fat*.[9] This impact on consumer behavior alone was assumed to take effect during the first year of implementation and no further reduction thereafter. For industry reformulation, Huang et al. assumed no reformulation in the first year of labeling implementation, then 8.25% (95% CI: 7.5% to 9.0%) of the sugar-containing products would be reformulated each of years 2 to 5 during the intervention period to achieve 25% reduction in added sugar content in these products, with no additional reformulation thereafter.[10-12] In sum, this represents an 8.25% x 4 years x 25% = 8.25% net reduction in added sugar amounts of U.S. sugar-containing products over the intervention period.[10]

© 2021 Du M et al. *JAMA Network Open.*

eAppendix 5. Methods of estimating policy implementation costs

We estimated the costs of implementing the Nutrition Facts added sugar labeling for both government and industry, including government administration cost, monitoring and evaluation costs, industry compliance costs and reformulation costs, based on FDA's budget report,[13] the Nutrition Review Project report,[14] and FDA's RIA[11] (e**Table 2**).

© 2021 Du M et al. *JAMA Network Open.*

eTable 2. Implementation cost estimates for the Nutrition Facts added sugar labeling policy (in 2015 US dollars)

Policy Impact	Cost Category	One-time Cost[a]	Annual Cost[a]	Source
Consumer behavior alone	1. Government administration[b]	$4,536,810 ($3,629,448 to $5,444,172)	N/A	FDA's budget report[1]
	2. Government monitoring and evaluation[b]	N/A	$251,824 ($201,459 to $302,189) (starting from 2nd year)	Nutrition Review Project report[2]
	3. Industry compliance	$1,667,070,000 ($842,100,000 to $2,972,530,000)	N/A	FDA's RIA[3]
Industry reformulation	4. Industry reformulation	N/A	$44,550,571 (20,041,035 to $82,669,271) (starting from 2nd year)	

[a] Policy intervention costs were inflated to 2015 US (December) dollars using the Consumer Price Index.
[b] Given no range of uncertainty was provided in source materials, we assumed 20% uncertainty around these costs.

Added sugar labeling is one of many provisions in FDA's rule to update the Nutrition Facts label. The cost of implementing all provisions is fixed for government (administration and monitoring and evaluation) and industry compliance. Therefore, we attributed 50% of the costs for implementing the entire labeling policy to the costs of added sugar labeling for government administration (#1), government monitoring and evaluation (#2), and industry compliance cost (#3) as this approach generated more conservative estimates. Uncertainty for the costs associated with government administration (#1) and government monitoring and evaluation (# 2) was not provided in the source materials.[13,14] We assumed that uncertainty is 20% around these costs.

For annual costs, namely the government monitoring and evaluation cost (#2) and the industry reformulation cost (#4), we applied 3% discounting rate recommended by the Second Panel on cost-effectiveness in health and medicine[15] to reflect the present value of future costs of government monitoring and evaluation and industry reformulation. The model is a closed cohort model, so we computed the discounted present value of per-person costs and total national costs for persons alive at implementation who remained alive in each subsequent year (not for the larger total US population in each year, which also has growth from immigration and new persons reaching the threshold age). The year-specific discounting factor is estimated by $1/(1+3\%)^{(t-1)}$ (t is number of years of policy intervention, t=1, 2, 3, …, lifetime) (e**Table 3**):

© 2021 Du M et al. *JAMA Network Open.*

eTable 3. Discounting factor in each year over a lifetime

Year	Discounting factor
1	1.000
2	0.971
3	0.943
4	0.915
5	0.888
⋮	⋮
67	0.142
68	0.138
69	0.134
70	0.130

© 2021 Du M et al. *JAMA Network Open.*

As our model estimated the costs and health outcomes based on a closed cohort and the population size decline over time, we need to express the annual costs in proportion to the population at risk. The population at risk was estimated based on the proportion of death ($P_{dt,}$ t=1, 2, 3, …) in each year. We first obtained the proportion of people who are alive at each year by calculating 1-P_{dt} (t=1, 2, 3, …). Then we multiplied the baseline population size of 235 million by the proportion of people who are alive in each year (e**Table 4**).

eTable 4. Population size of people who are alive in each year over a lifetime (in millions)

Year	Population Size (Million)
1	235.2
2	233.7
3	232.1
4	230.4
5	228.2
⋮	⋮
67	5.832
68	4.348
69	3.157
70	2.233

We then estimated the per-person annual cost for cost categories #2 and #4, by dividing the annual cost estimated in the second year of implementing the policy among all US population by the population size in the second year. Specifically, for government monitoring and evaluation, the per person annual cost is estimated $251,824/233,719,989=$0.00108, and that for industry reformulation is $44,550,571/233,719,989=$0.190615. Taken together, to estimate the discounted annual cost of #2 and #4, we multiplied the population at risk, the per person annual cost estimated at year-2, and the year-specific discounting factor, using: discounted annual cost = population at risk x per-person annual cost x $1/(1+3\%)^{(t-1)}$.

© 2021 Du M et al. *JAMA Network Open.*

eAppendix 6. Annual health-related costs among cancer patients and the general population without cancer

The annual health-related costs data include: 1) medical expenditure, 2) productivity loss form missed work days or disability, and 3) patient time cost associated with receiving care for cancer survivors by age (under 65 vs. above 65 years old) and phase of care (initial, continuing, end-year of life); 4) medical expenditure, 5) productivity loss, and 6) patient time cost for individuals without cancer by age and status of end year of life. The description of data source and data structure were provided in e**Table 5**.

© 2021 Du M et al. *JAMA Network Open.*

eTable 5. Description of Data Source of Health-Related Expenditures

	A. Cancer Survivors		B. Individuals without Cancer	
	Data source<br>(Excess or Total)	Category	Data source	Category
**Medical expenditure**	Mariotto et al. 2011, SEER-Medicare, in 2010 US dollars<br>(Excess)	-by phase of care[a]<br>-by age (under 65 vs. above 65 years old)<br>-by sex	Kim et al. 2018, MEPS 2013-2014, *in vivo* analysis, in 2014 US dollars<br>(Total)	-Medical expenditure among all US adults<br>-by 32 subgroups stratified by age, sex, and race/ethnicity
			Hogen et al. 2001, SEER-Medicare (65+), in 2001 US dollars<br>(Total)	-Medical expenditure in the end year of life among all US adults
**Productivity loss**	Zheng et al. 2016, MEPS 2008-2012, data available for colorectal, female breast, and prostate cancers, in 2012 US dollars<br>(Total)	-by age		
	Guy et al. 2013, MEPS 2008-2010, all types of cancer, in 2010 US dollars<br>(Total)	-by age<br>-by time interval since cancer diagnosis (less than 1 year vs. greater than 1 year)[b]	Guy et al. 2013, MEPS 2008-2010, in 2010 US dollars<br>(Total)	-by age
**Patient time cost**	Yabroff et al. 2014, MEPS 2008-2011, all types of cancer, in 2011 US dollars<br>(Total)	-by age	Yabroff et al. 2014, MEPS 2008-2011, in 2011 US dollars<br>(Total)	-by age

[a] The definition of phases of care: 1) initial phase, defined as the first 12 months following diagnosis, 2) end-year of life phase, defined as the final 12 months of life, and 3) the continuing phase, defined as all the months between the initial phase and the end-year of life. The costs of end-year of life varied by cause of death, either cancer-specific death or death due to other causes.
[b] Weighted means were calculated based on sample sizes and strata means.

We extracted the raw data for each of the costing component from the published literatures.[2,16-20] The overall assumptions for data extraction include: 1) health-related costs for breast cancer among post-menopausal females, advanced prostate cancer, esophageal adenocarcinoma, and stomach cardia cancer, by age, sex, and phase of cancer care, were the same as those for breast cancer, prostate cancer, esophagus cancer, and stomach cancer; 2) if no data available for a specific cancer type, we assumed the costs for that cancer type were the same as the estimates of costs for all-cancer sites, e.g., medical expenditure for all-cancer sites were used to replace the medical expenditures for multiple myeloma, gallbladder, liver, and thyroid cancers; 3) we extracted the costs for end-year of life due to cancer death and assumed that death due to other causes is not a competing outcome; 4) we assumed that the end-year life medical expenditure for individuals without cancer does not vary by the 32 subgroups.

If a specific costing component was not reported directly in the raw data, we calculated the cost for that component based on available data. For example, the annual productivity loss for colorectal cancer were reported as a percentage of total health-related costs.[20] We multiplied the percentage and the total health-related costs to obtain the productivity loss for colorectal cancer. We also performed data imputation for unavailable data. For instance, the annual productivity loss for all-cancer sites was reported by time interval since cancer diagnosis (diagnosed within one year vs. diagnosed greater than one year).[16] To obtain this costing component by the defined phases of care, we calculated the weighted means which was used as

© 2021 Du M et al. *JAMA Network Open.*

the annual productivity loss for continuing phase. We then assumed that the productivity loss in the initial phase and end-of-life phase of cancer care are 1.3 times and 4 times to the mean estimates based on available data for other cancers.[2,16] For individuals without cancer, we assumed that the end-of-life productivity loss is 4 times to the mean estimate of the productivity loss. The same rules applied to data imputation for patient time costs.

We then applied the age shifting to keep the expenditures consistent within each age group. Starting from 2021, individuals in the cohort of 55-64 years old will turn into the cohort of 65 years and older. Therefore, we assumed that starting from 2021, the heath-related expenditures for individuals who were in the cohort of 55-64 years old would be the same as those for individuals who were in the cohort of 65 years and older at the beginning of the DiCOM model. Based on the same assumption, starting from 2031 and 2047, the health-related expenditures for the cohort of 45-54 years old and those for the cohort of 20-44 years old were projected to be the same as those for the cohort of 65 years and older, respectively. We followed the same rule and applied the age shifting for the health-related expenditures for individuals without cancer. All estimations and projections were performed in SAS 9.4. All health-related expenditures were inflated to 2015 US dollars using the Personal Health Care (PHC) index.

© 2021 Du M et al. *JAMA Network Open.*

eTable 6. Defining population and 32 subgroups

Subgroups	Age	Sex	Race/Ethnicity
**1**	20-44y	Female	NHW
**2**	20-44y	Female	NHB
**3**	20-44y	Female	HISP
**4**	20-44y	Female	OTH
**5**	20-44y	Male	NHW
**6**	20-44y	Male	NHB
**7**	20-44y	Male	HISP
**8**	20-44y	Male	OTH
**9**	45-54y	Female	NHW
**10**	45-54y	Female	NHB
**11**	45-54y	Female	HISP
**12**	45-54y	Female	OTH
**13**	45-54y	Male	NHW
**14**	45-54y	Male	NHB
**15**	45-54y	Male	HISP
**16**	45-54y	Male	OTH
**17**	55-64y	Female	NHW
**18**	55-64y	Female	NHB
**19**	55-64y	Female	HISP
**20**	55-64y	Female	OTH
**21**	55-64y	Male	NHW
**22**	55-64y	Male	NHB
**23**	55-64y	Male	HISP
**24**	55-64y	Male	OTH
**25**	65+y	Female	NHW
**26**	65+y	Female	NHB
**27**	65+y	Female	HISP
**28**	65+y	Female	OTH
**29**	65+y	Male	NHW
**30**	65+y	Male	NHB
**31**	65+y	Male	HISP
**32**	65+y	Male	OTH

© 2021 Du M et al. *JAMA Network Open.*

eTable 7. Characteristics of US adults aged 20 years or older participated in the NHANES, 2013-2016

**Characteristics** (N=10058)		**Added Sugar Consumption**, g/day
**Age**, years	47.8 ± 0.41	
**Age groups**, years, N (%)		
20-44	4317 (44.5)	49.4 ± 0.50
25-54	1704 (18.4)	53.6 ± 1.05
55-64	1723 (17.3)	54.7 ± 0.74
≥65	2314 (19.9)	56.9 ± 0.69
**Sex**, N (%)		
Male	4826 (48.3)	47.1 ± 0.43
Female	5232 (51.7)	57.7 ± 0.52
**Race/ethnicity**, N (%)		
Non-Hispanic White	3940 (65.0)	53.3 ± 0.51
Non-Hispanic Black	2067 (11.2)	55.1 ± 0.90
Hispanic	2668 (14.9)	51.5 ± 0.64
Other	1383 (8.91)	46.2 ± 1.08
**Education**, N (%)		
Less than high school graduate	2177 (14.3)	56.6 ± 0.69
High school graduate	2248 (21.6)	55.9 ± 0.66
Some college	3067 (33.1)	54.2 ± 0.69
College graduate	2561 (31.0)	46.8 ± 0.57
**Family income to poverty ratio**, N (%)		
<1.30	3860 (28.3)	56.6 ± 0.79
1.30-1.84	2840 (26.7)	54.4 ± 0.63
1.85-2.99	1723 (20.4)	50.9 ± 0.84
≥3.00	1635 (24.5)	47.5 ± 0.77
**Body mass index (BMI)**, kg/m^2	29.3 ± 0.16	
Weight status, N (%)		
Underweight (BMI<18.5)	145 (1.36)	56.2 ± 2.18
Normal weight (BMI=18.5-24.9)	2670 (27.2)	52.3 ± 0.65
Overweight/Obese (BMI≥25)	7158 (71.4)	52.7 ± 0.42

© 2021 Du M et al. *JAMA Network Open.*

eTable 8. Consumption of added sugar from foods and beverages among US adults participated in 2013-2016 NHANES, by 32 subgroups

**Age group**, years	**Sex**	**Race/ethnicity**	**Baseline consumption**, g/day (mean ± SE)
20-44	Female	NHW	53.8 ± 0.93
		NHB	57.8 ± 2.02
		Hispanic	55.3 ± 0.94
		Other	49.0 ± 1.14
	Male	NHW	45.0 ± 0.97
		NHB	45.9 ± 1.41
		Hispanic	45.9 ± 0.88
		Other	39.9 ± 2.14
45-54	Female	NHW	60.9 ± 1.85
		NHB	61.0 ± 1.88
		Hispanic	54.4 ± 1.87
		Other	51.5 ± 3.81
	Male	NHW	48.5 ± 1.18
		NHB	46.9 ± 1.78
		Hispanic	48.4 ± 1.22
		Other	38.8 ± 2.05
55-64	Female	NHW	61.4 ± 1.57
		NHB	63.7 ± 1.96
		Hispanic	55.2 ± 1.08
		Other	52.0 ± 3.18
	Male	NHW	49.2 ± 1.49
		NHB	52.6 ± 1.01
		Hispanic	49.3 ± 1.80
		Other	39.5 ± 2.34
≥65	Female	NHW	61.5 ± 1.15
		NHB	66.5 ± 1.45
		Hispanic	60.6 ± 1.34
		Other	57.4 ± 2.47
	Male	NHW	50.9 ± 1.12
		NHB	56.0 ± 1.69
		Hispanic	50.0 ± 1.24
		Other	49.4 ± 2.87

© 2021 Du M et al. *JAMA Network Open.*

eTable 9 Relative risk estimates of etiologic relationships between body mass index (BMI) and cancer

Cancer Type	No. of Studies	No. of Events	Source	Evidence Grading	RR (95% CI) Per 5 kg/m^2	Statistical Heterogeneity
Corpus uteri	26	18,717	CUP, 2013	Convincing ↑risk	1.50 (1.42-1.59)	I^2=86.2% P<0.0001
Esophageal (adenocarcinoma)	9	1,725	CUP, 2016	Convincing ↑risk	1.48 (1.35-1.62)	I^2=36.7% P=0.13
Kidney	23	15,575	CUP, 2015	Convincing ↑risk	1.30 (1.25-1.35)	I^2=38.8% P=0.03
Liver	12	14, 311	CUP, 2015	Convincing ↑risk	1.30 (1.16-1.46)	I^2=78.3% P=0.000
Gallbladder	8	6,004	CUP, 2015	Probable ↑risk	1.25 (1.15-1.37)	I^2=52.3% P=0.04
Stomach (cardia)	7	2,050	CUP, 2016	Probable ↑risk	1.23 (1.07-1.40)	I^2=55.6% P=0.04
Breast (post-menopausal)	56	80,404	CUP, 2017	Convincing ↑risk	1.12 (1.09-1.15)	I^2=75% P<0.001
Pancreas	23	9,504	CUP, 2011	Convincing ↑risk	1.10 (1.07-1.14)	I^2=19% P=0.20
Multiple myeloma	20	1,388	IARC, 2016[22]	Sufficient (IRAC) ↑risk	1.09 (1.03-1.16)	Not reported
Prostate (advanced)	24	11,149	CUP, 2014	Probable ↑risk	1.08 (1.04-1.12)	I^2=18.8% P=0.21
Thyroid	22	3,100	IARC, 2016[22]	Sufficient (IARC) ↑risk	1.06 (1.02-1.10)	Not reported
Ovary	25	15,899	CUP, 2013	Probable ↑risk	1.06 (1.02-1.11)	I^2=55.1% P=0.001
Colorectal	38	71,089	CUP, 2017	Convincing ↑risk	1.05 (1.03-1.07)	I^2=74.2% P=0.000

© 2021 Du M et al. *JAMA Network Open.*

eTable 10. Health-related quality of life among US cancer patients aged 20 years or older, by cancer type and phase of care

Cancer Type	Cancer Phase	Health Related Quality of Life	Source
		mean (SE)	
Endometrial	Overall	0.80 (0.14)	Naik et al.[23]
Esophageal Adenocarcinoma	Overall	0.69 (0.26)	Wildi et al.[24]
Kidney	Overall	0.78 (0.14)	Pickard et al.[25]
Liver	Overall	0.79 (0.19)	Naik et al.[23]
Gallbladder	Overall	0.79 (0.19)	Naik et al.[23]
Stomach (gastric cardia)	Initial:<br>Continuous:<br>End of Life:	0.84 (0.25)<br>0.86 (0.24)<br>0.65 (0.33)	Zhou et al.[26]
Female Breast (post-menopausal)	Initial:<br>Continuous:<br>End of Life:	0.78 (0.19)<br>0.81 (0.20)<br>0.64 (0.16)	Yabroff et al.[27]
Pancreas	Overall	0.65 (0.30)	Müller-Nordhorn et al.[28]
Multiple myeloma	Overall	0.79 (0.19)	Naik et al.[23]
Advanced Prostate	Initial:<br>Continuous:<br>End of Life:	0.78 (0.20)<br>0.76 (0.19)<br>0.59 (0.15)	Yabroff et al.[27]
Thyroid	Overall	0.85 (0.13)	Naik et al.[23]
Ovary	Overall	0.77 (0.17)	Pickard et al.[25]
Colorectal	Initial:<br>Continuous:<br>End of Life:	0.76 (0.19)<br>0.84 (0.20)<br>0.64 (0.26)	Färkkilä et al.[29]

© 2021 Du M et al. *JAMA Network Open.*

eTable 11. Baseline medical costs, productivity loss, and patient time costs among US cancer patients aged 20 years or older, by cancer type

Cancer type	Sex	Age	Medical costs			Productivity loss			Patient time cost		
			Initial	Continuous	End-of-life	Initial	Continuous	End-of-life	Initial	Continuous	End-of-life
Esophageal Adenocarcinoma	Female	<65	95439	6853	156417	4884	3757	15027	650	500	2001
		≥65	79532	6853	104278	6984	5372	21489	1187	913	3652
	Male	<65	95787	6450	155612	4884	3757	15027	650	500	2001
		≥65	79822	6450	103742	6984	5372	21489	1187	913	3652
Stomach (Gastric Cardia)	Female	<65	85291	3977	155636	4884	3757	15027	650	500	2001
		≥65	71076	3977	103758	6984	5372	21489	1187	913	3652
	Male	<65	94144	4282	160695	4884	3757	15027	650	500	2001
		≥65	78453	4282	107130	6984	5372	21489	1187	913	3652
Liver	Female	<65	40173	5859	95782	4884	3757	15027	650	500	2001
		≥65	40173	5859	95782	6984	5372	21489	1187	913	3652
	Male	<65	41161	7363	97473	4884	3757	15027	650	500	2001
		≥65	41161	7363	97473	6984	5372	21489	1187	913	3652
Pancreatic	Female	<65	112154	8672	164911	4884	3757	15027	650	500	2001
		≥65	93462	8672	109941	6984	5372	21489	1187	913	3652
	Male	<65	112911	11697	169673	4884	3757	15027	650	500	2001
		≥65	94092	11697	113115	6984	5372	21489	1187	913	3652
Advanced Prostate	Male	<65	23652	3201	93363	3715	2858	11432	650	500	2001
		≥65	19710	3201	62242	6549	5038	20152	1187	913	3652
Colorectal	Female	<65	61593	3159	126778	10330	7946	31784	650	500	2001

© 2021 Du M et al. *JAMA Network Open.*

		≥65	51327	3159	84519	7479	5753	23012	1187	913	3652
	Male	<65	62174	4595	128507	10330	7946	31784	650	500	2001
		≥65	51812	4595	85671	7479	5753	23012	1187	913	3652
Endometrial	Female	<65	32129	1535	105262	4884	3757	15027	650	500	2001
		≥65	26775	1535	70175	6984	5372	21489	1187	913	3652
Ovarian	Female	<65	98788	8296	149573	4884	3757	15027	650	500	2001
		≥65	82324	8296	99715	6984	5372	21489	1187	913	3652
Gallbladder	Female	<65	40173	5859	95782	4884	3757	15027	650	500	2001
		≥65	40173	5859	95782	6984	5372	21489	1187	913	3652
	Male	<65	41161	7363	97473	4884	3757	15027	650	500	2001
		≥65	41161	7363	97473	6984	5372	21489	1187	913	3652
Kidney (Renal Cell)	Female	<65	46077	6255	110765	4884	3757	15027	650	500	2001
		≥65	38397	6255	73843	6984	5372	21489	1187	913	3652
	Male	<65	46048	6018	117123	4884	3757	15027	650	500	2001
		≥65	38374	6018	78082	6984	5372	21489	1187	913	3652
Breast (Postmenopausal)	Female	<65	27693	2207	94284	5985	4604	18416	650	500	2001
		≥65	23078	2207	62856	4752	3655	14620	1187	913	3652
Thyroid	Female	<65	40173	5859	95782	4884	3757	15027	650	500	2001
		≥65	40173	5859	95782	6984	5372	21489	1187	913	3652
	Male	<65	41161	7363	97473	4884	3757	15027	650	500	2001
		≥65	41161	7363	97473	6984	5372	21489	1187	913	3652

© 2021 Du M et al. *JAMA Network Open.*

Multiple Myeloma	Female	<65	40173	5859	95782	4884	3757	15027	650	500	2001
		≥65	40173	5859	95782	6984	5372	21489	1187	913	3652
	Male	<65	41161	7363	97473	4884	3757	15027	650	500	2001
		≥65	41161	7363	97473	6984	5372	21489	1187	913	3652

© 2021 Du M et al. *JAMA Network Open.*

eTable 12. Baseline medical costs, productivity loss, and patient time costs among general population aged 20 years or older in the US, by 32 subgroups

Age group, years	Sex	Race/ethnicity	Medical costs		Productivity loss		Patient time cost	
			Annual general costs	End-of-life costs	Annual general costs	End-of-life costs	Annual general costs	End-of-life costs
20-44	Female	NHW	4020	40000	2040	8160	226	904
		NHB	3100	40000	2040	8160	226	904
		Hispanic	2355	40000	2040	8160	226	904
		Other	2617	40000	2040	8160	226	904
	Male	NHW	2022	40000	2040	8160	226	904
		NHB	2279	40000	2040	8160	226	904
		Hispanic	1145	40000	2040	8160	226	904
		Other	1803	40000	2040	8160	226	904
45-54	Female	NHW	5371	40000	2040	8160	226	904
		NHB	5712	40000	2040	8160	226	904
		Hispanic	3196	40000	2040	8160	226	904
		Other	4082	40000	2040	8160	226	904
	Male	NHW	3812	40000	2040	8160	226	904
		NHB	3639	40000	2040	8160	226	904
		Hispanic	3612	40000	2040	8160	226	904
		Other	2560	40000	2040	8160	226	904
55-64	Female	NHW	7300	40000	2040	8160	226	904
		NHB	5479	40000	2040	8160	226	904
		Hispanic	4607	40000	2040	8160	226	904
		Other	3951	40000	2040	8160	226	904
	Male	NHW	6519	40000	2040	8160	226	904
		NHB	6455	40000	2040	8160	226	904
		Hispanic	5077	40000	2040	8160	226	904
		Other	6320	40000	2040	8160	226	904
≥65	Female	NHW	8997	40000	4409	8160	607	904
		NHB	9585	40000	4409	8160	607	904
		Hispanic	8847	40000	4409	8160	607	904
		Other	8625	40000	4409	8160	607	904

© 2021 Du M et al. *JAMA Network Open.*

	Male	NHW	9334	40000	4409	8160	607	904
		NHB	7367	40000	4409	8160	607	904
		Hispanic	5640	40000	4409	8160	607	904
		Other	7461	40000	4409	8160	607	904

© 2021 Du M et al. *JAMA Network Open.*

eTable 13. Estimated changes in health-related costs associated with Nutrition Facts added sugar labeling on reducing cancer burdens in the US over a lifetime, by cancer type[a]

**Changes in Health-Related Costs[b]** ($, millions)	Added Sugar Labeling Policy	
	**Consumer Behavior** Median (2.5% to 97.5%)	**Consumer Behavior + Industry Response** Median (2.5% to 97.5%)
Medical Cost		
Kidney Cancer (Renal Cell)	-347 (-439 to -263)	-698 (-850 to -561)
Liver Cancer	-346 (-450 to -266)	-684 (-863 to -549)
Breast Cancer (Postmenopausal)	-226 (-351 to -128)	-544 (-747 to -392)
Endometrial Cancer	-218 (-334 to -126)	-536 (-708 to -390)
Pancreatic Cancer	-173 (-226 to -126)	-368 (-455 to -289)
Esophageal Adenocarcinoma	-155 (-213 to -112)	-300 (-384 to -224)
Colorectal Cancer	-96.0 (-122 to -72.1)	-192 (-236 to -152)
Stomach Cancer (Gastric Cardia)	-64.3 (-95.3 to -44.5)	-127 (-174 to -91.1)
Thyroid Cancer	-50.1 (-76.2 to -32.9)	-110 (-155 to -79.9)
Multiple Myeloma	-46.2 (-65 to -32.6)	-98.3 (-129 to -73.4)
Gallbladder Cancer	-21.2 (-27.0 to -16.0)	-45.3 (-55.2 to -37.0)
Ovarian Cancer	-20.6 (-35.8 to -9.33)	-50.9 (-76.9 to -29.0)
Advanced Prostate Cancer	-14.8 (-20.6 to -10.3)	-28.0 (-37.6 to -20.2)
Productivity Loss		
Endometrial Cancer	-196 (-301 to -109)	-484 (-641 to -344)
Kidney Cancer (Renal Cell)	-156 (-199 to -119)	-314 (-383 to -252)
Breast Cancer (Postmenopausal)	-99.8 (-163 to -52.2)	-246 (-340 to -172)
Liver Cancer	-83.5 (-109 to -64.4)	-166 (-207 to -134)
Colorectal Cancer	-43.6 (-55.5 to -32.4)	-88.1 (-109 to -69.5)
Thyroid Cancer	-27.4 (-42.9 to -17.4)	-61.2 (-87.9 to -43.8)
Esophageal Adenocarcinoma	-26.5 (-37.0 to -19.0)	-51.8 (-67.3 to -39.3)
Pancreatic Cancer	-25.7 (-34.0 to -18.6)	-54.8 (-67.3 to -43.1)
Multiple Myeloma	-16.1 (-23.0 to -11.2)	-34.5 (-45.6 to -25.7)
Stomach Cancer (Gastric Cardia)	-12.1 (-17.7 to -8.38)	-24.2 (-33.0 to -17.4)
Advanced Prostate Cancer	-9.31 (-13.1 to -6.37)	-17.7 (-24.3 to -12.5)
Gallbladder Cancer	-5.46 (-7.02 to -4.14)	-11.7 (-14.2 to -9.58)
Ovarian Cancer	-4.68 (-8.27 to -2.12)	-11.6 (-17.9 to -6.38)
Patient Time Cost		
Endometrial Cancer	-31.4 (-48.6 to -17.3)	-77.4 (-102 to -55.3)
Kidney Cancer (Renal Cell)	-24.7 (-31.5 to -18.8)	-49.9 (-60.4 to -40.1)
Breast Cancer (Postmenopausal)	-22.7 (-36.3 to -12.3)	-54.8 (-76.6 to -37.3)
Liver Cancer	-13.1 (-16.9 to -10.1)	-26.1 (-32.3 to -21.0)
Colorectal Cancer	-5.23 (-6.65 to -3.89)	-10.6 (-13.0 to -8.37)
Thyroid Cancer	-4.24 (-6.54 to -2.73)	-9.47 (-13.5 to -6.77)
Esophageal Adenocarcinoma	-4.22 (-5.96 to -3.04)	-8.24 (-10.7 to -6.26)
Pancreatic Cancer	-4.12 (-5.42 to -3.00)	-8.74 (-10.7 to -6.86)
Multiple Myeloma	-2.59 (-3.67 to -1.80)	-5.53 (-7.36 to -4.11)
Stomach Cancer (Gastric Cardia)	-1.92 (-2.81 to -1.33)	-3.85 (-5.27 to -2.79)
Advanced Prostate Cancer	-1.71 (-2.41 to -1.18)	-3.25 (-4.45 to -2.31)
Gallbladder Cancer	-0.88 (-1.13 to -0.67)	-1.88 (-2.29 to -1.54)
Ovarian Cancer	-0.74 (-1.29 to -0.34)	-1.81 (-2.78 to -1.00)

[a]Values are the median estimates (95% uncertainty intervals) of each distribution of 1000 simulations.
[b] Health-related costs were inflated to 2015 US dollars using the Personal Health Care (PHC) index. Negative costs represent savings. Costs are medians from 1000 simulations so may not add up to totals.

© 2021 Du M et al. *JAMA Network Open.*

eTable 14. Estimated new cancer cases averted by Nutrition Facts added sugar labeling policy in the US by age, gender, race/ethnicity, and cancer type over a lifetime (US population=235,162,844)[a]

Cancer Type	Policy Scenario	20-44 y		45-54 y		55-64 y		65 + y	
		Female	Male	Female	Male	Female	Male	Female	Male
**Endometrial**									
Age	*consumer behavior*	3040 (1060 to 5750)		614 (-468 to 2210)		1170 (410 to 2470)		1080 (505 to 2050)	
	+industry response	7660 (4210 to 11900)		2470 (857 to 4790)		2620 (1250 to 4580)		2230 (1140 to 3720)	
Race/Ethnicity									
Non-Hispanic White	*consumer behavior*	1680 (196 to 4060)	0	507 (-472 to 1940)	0	778 (79 to 2020)	0	846 (323 to 1810)	0
	+industry response	4310 (1520 to 7980)	0	1750 (162 to 3850)	0	1840 (590 to 3780)	0	1710 (658 to 3250)	0
Non-Hispanic Black	*consumer behavior*	635 (-82 to 1830)	0	-1 (-248 to 443)	0	215 (47 to 498)	0	124 (36 to 297)	0
	+industry response	1440 (412 to 3020)	0	322 (-81 to 896)	0	421 (152 to 812)	0	270 (91 to 525)	0
Hispanic	*consumer behavior*	408 (-185 to 1320)	0	4 (-199 to 377)	0	86 (6 to 230)	0	58 (-4 to 166)	0
	+industry response	1330 (130 to 2820)	0	252 (-70.2 to 795)	0	215 (52 to 441)	0	150 (31 to 319)	0
Other	*consumer behavior*	202 (69 to 425)	0	53 (-53 to 225)	0	43 (-7 to 123)	0	25 (-3 to 78)	0
	+industry response	418 (176 to 752)	0	112 (10 to 305)	0	81 (26 to 177)	0	53 (16 to 117)	0
**Breast (Postmenopausal)**									
Age	*consumer behavior*	2450 (856 to 4770)		586 (-427 to 2160)		1200 (412 to 2610)		1210 (532 to 2430)	
	+industry response	6070 (3220 to 9930)		2210 (700 to 4620)		2720 (1270 to 4700)		2510 (1220 to 4390)	
Race/Ethnicity									
Non-Hispanic White	*consumer behavior*	1450 (142 to 3640)	0	378 (-507 to 1880)	0	847 (87 to 2250)	0	980 (362 to 2210)	0
	+industry response	3660	0	1520 (100 to 3710)	0	2020 (640 to 3980)	0	2030	0

© 2021 Du M et al. *JAMA Network Open.*

		(1150 to 7420)						(782 to 3990)	
Non-Hispanic Black	*consumer behavior*	441 (-61 to 1330)	0	76 (-119 to 447)	0	196 (48 to 440)	0	114 (29 to 291)	0
	+industry response	1020 (277 to 2330)	0	333 (10 to 863)	0	375 (140 to 734)	0	249 (78 to 522)	0
Hispanic	*consumer behavior*	251 (-137 to 887)	0	-5 (-157 to 300)	0	75 (6 to 208)	0	55 (-4 to 166)	0
	+industry response	868 (78 to 1910)	0	186 (-66 to 595)	0	187 (44 to 384)	0	143 (32 to 305)	0
Other	*consumer behavior*	189 (61 to 409)	0	59 (-58 to 263)	0	56 (-7 to 166)	0	35 (-4 to 109)	0
	+industry response	395 (153 to 723)	0	124 (13 to 363)	0	105 (34 to 242)	0	75 (22 to 167)	0
**Kidney (Renal Cell)**									
Age	*consumer behavior*	3180 (2210 to 4600)		778 (304 to 1410)		865 (483 to 1450)		672 (379 to 1120)	
	+industry response	5960 (4350 to 8110)		1890 (1130 to 2820)		1740 (1140 to 2570)		1360 (785 to 2060)	
Race/Ethnicity									
Non-Hispanic White	*consumer behavior*	349 (53 to 842)	1750 (1110 to 2990)	93 (-103 to 389)	350 (25 to 904)	177 (18 to 476)	409 (124 to 949)	200 (74 to 430)	297 (75 to 695)
	+industry response	887 (308 to 1710)	2800 (1590 to 4640)	347 (28 to 772)	826 (237 to 1670)	418 (129 to 839)	803 (296 to 1560)	416 (154 to 783)	615 (181 to 1230)
Non-Hispanic Black	*consumer behavior*	144 (-17 to 407)	214 (9 to 563)	4 (-54 to 104)	104 (25 to 254)	55 (11 to 130)	69 (27 to 140)	34 (9 to 82)	41 (12 to 86)
	+industry response	324 (87 to 706)	467 (137 to 971)	77 (-15 to 213)	191 (77 to 376)	107 (39 to 212)	134 (53 to 238)	73 (24 to 145)	74 (30 to 138)
Hispanic	*consumer behavior*	114 (-62 to 367)	340 (78 to 764)	0 (-60 to 112)	131 (45 to 270)	28 (2 to 74)	50 (-10 to 152)	21 (-1 to 57)	32 (9 to 70)
	+industry response	395 (27 to 812)	763 (177 to 1430)	76 (-19 to 231)	253 (96 to 451)	71 (17 to 145)	111 (25 to 242)	53 (11 to 112)	64 (22 to 118)
Other	*consumer behavior*	32 (11 to 66)	83 (5 to 217)	10 (-9 to 42)	25 (2 to 57)	9 (-1 to 27)	24 (7 to 50)	6 (-1 to 18)	12 (0 to 30)
	+industry response	66 (28 to 120)	150 (61 to 315)	20 (2 to 56)	43 (17 to 82)	17 (6 to 39)	36 (18 to 67)	13 (4 to 28)	20 (7 to 42)
**Liver**									
Age	*consumer behavior*	2990 (2070 to 4450)		841 (380 to 1500)		717 (422 to 1190)		476 (281 to 808)	

© 2021 Du M et al. *JAMA Network Open.*

	+industry response	5700 (4110 to 8320)		1920 (1110 to 2990)		1390 (917 to 2100)		958 (571 to 1510)	
Race/Ethnicity									
Non-Hispanic White	*consumer behavior*	223 (27 to 647)	1600 (1000 to 2950)	64 (-59 to 289)	307 (4 to 900)	100 (8 to 297)	315 (112 to 727)	106 (38 to 265)	195 (55 to 465)
	+industry response	569 (158 to 1300)	2540 (1330 to 4770)	222 (18 to 567)	749 (160 to 1720)	235 (74 to 528)	588 (224 to 1210)	222 (74 to 482)	400 (112 to 842)
Non-Hispanic Black	*consumer behavior*	115 (-16 to 363)	297 (17 to 853)	21 (-26 to 117)	148 (25 to 397)	33 (6 to 89)	91 (32 to 201)	20 (5 to 53)	52 (18 to 115)
	+industry response	261 (57 to 629)	646 (168 to 1410)	81 (-1 to 214)	281 (105 to 644)	68 (23 to 150)	179 (61 to 355)	43 (13 to 98)	96 (39 to 189)
Hispanic	*consumer behavior*	88 (-77 to 364)	403 (97 to 966)	2 (-56 to 137)	181 (76 to 372)	29 (0 to 95)	56 (-13 to 181)	21 (-2 to 67)	29 (5 to 77)
	+industry response	331 (-15 to 818)	892 (222 to 1840)	78 (-21 to 278)	325 (125 to 664)	73 (13 to 175)	123 (25 to 302)	56 (11 to 129)	62 (18 to 135)
Other	*consumer behavior*	35 (11 to 86)	96 (3 to 285)	15 (-9 to 56)	35 (1 to 83)	13 (-2 to 38)	36 (13 to 77)	8 (-2 to 28)	18 (2 to 45)
	+industry response	73 (27 to 156)	177 (63 to 424)	28 (5 to 79)	60 (23 to 122)	24 (7 to 56)	52 (27 to 106)	18 (5 to 44)	30 (12 to 63)
**Pancreatic**									
Age	*consumer behavior*	698 (402 to 1080)		207 (61 to 421)		290 (149 to 498)		251 (140 to 429)	
	+industry response	1450 (954 to 2070)		550 (306 to 883)		591 (368 to 914)		510 (295 to 793)	
Race/Ethnicity									
Non-Hispanic White	*consumer behavior*	114 (7 to 324)	311 (153 to 652)	27 (-50 to 152)	95 (10 to 273)	76 (6 to 224)	117 (30 to 295)	97 (38 to 224)	91 (23 to 216)
	+industry response	302 (85 to 648)	558 (267 to 1070)	122 (-4 to 310)	228 (64 to 500)	181 (45 to 396)	235 (76 to 490)	201 (77 to 414)	193 (55 to 387)
Non-Hispanic Black	*consumer behavior*	50 (-6 to 157)	38 (2 to 106)	-3 (-24 to 42)	20 (4 to 54)	23 (5 to 55)	16 (7 to 34)	15 (4 to 39)	10 (3 to 20)
	+industry response	114 (27 to 263)	84 (23 to 185)	25 (-10 to 91)	38 (14 to 83)	44 (17 to 92)	31 (12 to 60)	33 (10 to 73)	18 (7 to 34)
Hispanic	*consumer behavior*	32 (-14 to 113)	67 (14 to 159)	6 (-12 to 45)	25 (7 to 58)	9 (0 to 28)	12 (-2 to 36)	8 (-1 to 24)	8 (2 to 18)
	+industry response	105 (7 to 248)	147 (35 to 309)	28 (0 to 86)	50 (18 to 101)	25 (5 to 57)	27 (6 to 60)	21 (5 to 45)	15 (5 to 31)
Other	*consumer behavior*	15 (5 to 36)	19 (-1 to 61)	6 (-5 to 23)	8 (2 to 19)	6 (-1 to 19)	9 (3 to 18)	4 (-1 to 13)	4 (0 to 11)
	+industry response	31 (11 to 63)	36 (13 to 86)	12 (2 to 32)	14 (6 to 28)	11 (4 to 27)	13 (7 to 25)	9 (3 to 20)	7 (3 to 15)

© 2021 Du M et al. *JAMA Network Open.*

**Esophageal Adenocarcinoma**									
Age	*consumer behavior*	871 (587 to 1400)		230 (72 to 496)		265 (119 to 537)		204 (93 to 402)	
	+industry response	1530 (1010 to 2300)		545 (260 to 945)		523 (281 to 909)		421 (194 to 724)	
Race/Ethnicity									
Non-Hispanic White	*consumer behavior*	50 (4 to 127)	679 (412 to 1180)	15 (-16 to 63)	161 (13 to 422)	29 (3 to 78)	194 (61 to 465)	32 (11 to 73)	149 (41 to 339)
	+industry response	128 (39 to 249)	1100 (601 to 1840)	54 (5 to 122)	385 (117 to 775)	67 (21 to 138)	385 (147 to 757)	68 (24 to 129)	309 (89 to 605)
Non-Hispanic Black	*consumer behavior*	8 (-1 to 23)	15 (1 to 40)	0 (-3 to 7)	8 (2 to 20)	4 (1 to 9)	5 (2 to 11)	2 (1 to 6)	4 (1 to 8)
	+industry response	18 (5 to 39)	32 (10 to 68)	4 (-1 to 14)	15 (6 to 31)	7 (3 to 15)	11 (4 to 19)	5 (2 to 11)	7 (3 to 13)
Hispanic	*consumer behavior*	13 (-5 to 42)	74 (17 to 168)	1 (-6 to 13)	32 (13 to 64)	3 (0 to 9)	12 (-2 to 36)	2 (0 to 7)	7 (2 to 17)
	+industry response	42 (4 to 88)	162 (39 to 310)	9 (-2 to 27)	59 (24 to 107)	8 (2 to 17)	26 (6 to 60)	6 (2 to 14)	15 (5 to 29)
Other	*consumer behavior*	6 (2 to 13)	13 (1 to 34)	2 (-2 to 8)	5 (1 to 11)	2 (0 to 6)	5 (1 to 10)	1 (0 to 4)	2 (0 to 6)
	+industry response	12 (5 to 23)	23 (9 to 49)	4 (1 to 11)	8 (3 to 15)	4 (1 to 8)	7 (3 to 13)	3 (1 to 6)	4 (1 to 9)
**Colorectal**									
Age	*consumer behavior*	643 (433 to 934)		148 (54 to 281)		204 (112 to 349)		188 (106 to 317)	
	+industry response	1200 (812 to 1690)		365 (209 to 589)		403 (255 to 638)		380 (219 to 599)	
Race/Ethnicity									
Non-Hispanic White	*consumer behavior*	84 (2 to 269)	334 (213 to 577)	18 (-27 to 112)	59 (2 to 169)	52 (3 to 155)	83 (30 to 204)	77 (29 to 175)	64 (16 to 152)
	+industry response	216 (42 to 540)	521 (278 to 893)	78 (-4 to 220)	142 (33 to 328)	123 (30 to 284)	158 (60 to 335)	161 (58 to 324)	133 (38 to 284)
Non-Hispanic Black	*consumer behavior*	24 (-4 to 87)	33 (2 to 100)	-3 (-14 to 21)	16 (3 to 44)	13 (3 to 33)	13 (5 to 29)	9 (2 to 27)	9 (3 to 21)
	+industry response	59 (10 to 156)	73 (18 to 173)	11 (-9 to 50)	30 (10 to 70)	25 (8 to 56)	26 (9 to 53)	20 (6 to 49)	17 (6 to 34)
Hispanic	*consumer behavior*	27 (-4 to 93)	62 (11 to 161)	2 (-9 to 30)	27 (11 to 57)	7 (0 to 20)	10 (-2 to 34)	5 (-1 to 17)	7 (2 to 17)
	+industry response	75 (10 to 189)	136 (28 to 301)	17 (-3 to 58)	49 (19 to 98)	16 (3 to 39)	23 (5 to 57)	14 (2 to 33)	15 (5 to 30)

© 2021 Du M et al. *JAMA Network Open.*

Other	*consumer behavior*	12 (4 to 29)	24 (0 to 68)	4 (-4 to 16)	8 (0 to 18)	4 (-1 to 13)	7 (2 to 16)	3 (0 to 10)	4 (0 to 10)
	+industry response	25 (8 to 53)	43 (17 to 100)	8 (1 to 23)	13 (5 to 27)	7 (2 to 18)	11 (5 to 21)	6 (2 to 15)	7 (3 to 15)
**Thyroid**									
Age	*consumer behavior*	392 (205 to 690)		57 (-4 to 165)		76 (37 to 143)		58 (27 to 111)	
	+industry response	846 (479 to 1360)		177 (72 to 365)		155 (79 to 269)		120 (57 to 216)	
Race/Ethnicity									
Non-Hispanic White	*consumer behavior*	105 (-17 to 368)	122 (68 to 248)	11 (-27 to 107)	19 (-1 to 66)	26 (0 to 90)	24 (9 to 64)	26 (4 to 77)	16 (4 to 40)
	+industry response	282 (7 to 709)	204 (81 to 415)	62 (-16 to 219)	48 (4 to 128)	62 (5 to 174)	44 (15 to 107)	59 (8 to 145)	32 (7 to 74)
Non-Hispanic Black	*consumer behavior*	28 (-4 to 120)	7 (0 to 25)	4 (-4 to 26)	2 (0 to 8)	6 (1 to 18)	2 (1 to 4)	4 (1 to 12)	1 (0 to 3)
	+industry response	67 (1 to 211)	15 (2 to 45)	14 (-4 to 49)	5 (1 to 14)	12 (2 to 31)	3 (1 to 8)	8 (1 to 21)	2 (1 to 5)
Hispanic	*consumer behavior*	33 (-21 to 150)	21 (3 to 58)	-6 (-17 to 28)	6 (2 to 15)	4 (-1 to 15)	2 (0 to 8)	3 (0 to 12)	1 (0 to 3)
	+industry response	108 (-12 to 335)	45 (7 to 108)	9 (-15 to 60)	12 (3 to 27)	10 (0 to 30)	5 (1 to 14)	8 (1 to 23)	3 (1 to 6)
Other	*consumer behavior*	22 (5 to 59)	15 (4 to 39)	3 (-4 to 20)	3 (1 to 8)	3 (0 to 10)	2 (0 to 5)	2 (0 to 6)	1 (0 to 3)
	+industry response	46 (10 to 111)	24 (10 to 57)	8 (0 to 28)	5 (2 to 12)	5 (1 to 15)	3 (1 to 6)	3 (1 to 9)	2 (1 to 4)
**Multiple Myeloma**									
Age	*consumer behavior*	289 (128 to 511)		98 (28 to 212)		148 (85 to 263)		106 (54 to 193)	
	+industry response	648 (340 to 1030)		252 (123 to 444)		283 (170 to 473)		215 (111 to 370)	
Race/Ethnicity									
Non-Hispanic White	*consumer behavior*	37 (-12 to 147)	91 (24 to 270)	17 (-9 to 81)	36 (0 to 134)	26 (0 to 95)	65 (29 to 158)	33 (9 to 90)	35 (5 to 109)
	+industry response	103 (-7 to 297)	192 (44 to 509)	50 (-7 to 158)	91 (13 to 255)	63 (6 to 168)	115 (43 to 256)	67 (17 to 167)	76 (12 to 192)
Non-Hispanic Black	*consumer behavior*	34 (-5 to 137)	33 (0 to 115)	-5 (-20 to 36)	18 (4 to 53)	18 (6 to 52)	12 (2 to 33)	10 (1 to 31)	10 (4 to 24)
	+industry response	80 (1 to 243)	72 (10 to 202)	14 (-18 to 73)	35 (11 to 86)	35 (9 to 86)	24 (4 to 63)	22 (3 to 59)	18 (6 to 41)

© 2021 Du M et al. *JAMA Network Open.*

Hispanic	*consumer behavior*	2<br>(-19 to 49)	37<br>(5 to 111)	-5<br>(-12 to 15)	12<br>(3 to 37)	4<br>(-1 to 13)	6<br>(-2 to 23)	2<br>(-1 to 12)	4<br>(1 to 11)
	+industry response	32<br>(-18 to 122)	82<br>(9 to 213)	5<br>(-9 to 38)	26<br>(6 to 68)	10<br>(0 to 25)	14<br>(2 to 41)	7<br>(0 to 23)	8<br>(2 to 20)
Other	*consumer behavior*	4<br>(1 to 12)	11<br>(3 to 32)	2<br>(-1 to 7)	3<br>(0 to 9)	2<br>(0 to 5)	4<br>(2 to 9)	1<br>(0 to 3)	2<br>(0 to 5)
	+industry response	9<br>(1 to 21)	19<br>(7 to 48)	3<br>(0 to 10)	6<br>(2 to 14)	3<br>(1 to 8)	5<br>(3 to 12)	2<br>(0 to 6)	3<br>(1 to 7)
**Stomach (Gastric Cardia)**									
Age	*consumer behavior*	370 (237 to 666)		87 (21 to 237)		110 (46 to 253)		90 (40 to 191)	
	+industry response	665 (397 to 1150)		227 (88 to 465)		224 (102 to 442)		181 (81 to 347)	
Race/Ethnicity									
Non-Hispanic White	*consumer behavior*	25<br>(-2 to 87)	278<br>(165 to 552)	1<br>(-15 to 35)	64<br>(6 to 213)	15<br>(1 to 48)	72<br>(15 to 206)	18<br>(6 to 44)	58<br>(14 to 161)
	+industry response	66<br>(8 to 172)	443<br>(211 to 914)	21<br>(-9 to 76)	159<br>(31 to 390)	34<br>(6 to 89)	150<br>(38 to 369)	37<br>(11 to 80)	119<br>(25 to 290)
Non-Hispanic Black	*consumer behavior*	5<br>(-1 to 22)	10<br>(0 to 32)	0<br>(-2 to 6)	5<br>(1 to 14)	2<br>(1 to 6)	3<br>(1 to 8)	1<br>(0 to 4)	2<br>(0 to 5)
	+industry response	13<br>(2 to 37)	22<br>(4 to 56)	3<br>(-2 to 12)	9<br>(3 to 23)	4<br>(1 to 9)	7<br>(2 to 15)	3<br>(1 to 7)	4<br>(1 to 9)
Hispanic	*consumer behavior*	2<br>(-8 to 26)	25<br>(4 to 72)	-1<br>(-5 to 8)	9<br>(2 to 24)	2<br>(0 to 6)	4<br>(-1 to 15)	1<br>(0 to 5)	3<br>(1 to 8)
	+industry response	18<br>(-6 to 61)	57<br>(9 to 133)	3<br>(-3 to 17)	19<br>(4 to 41)	4<br>(0 to 11)	9<br>(2 to 26)	3<br>(0 to 9)	6<br>(1 to 14)
Other	*consumer behavior*	4<br>(1 to 11)	9<br>(0 to 26)	1<br>(-1 to 6)	2<br>(-1 to 7)	1<br>(0 to 5)	4<br>(2 to 9)	1<br>(0 to 3)	2<br>(0 to 5)
	+industry response	8<br>(2 to 19)	16<br>(5 to 39)	3<br>(0 to 9)	5<br>(1 to 12)	2<br>(1 to 7)	6<br>(3 to 11)	2<br>(0 to 5)	3<br>(1 to 7)
**Gallbladder**									
Age	*consumer behavior*	148 (89 to 225)		47 (17 to 84)		62 (37 to 100)		51 (30 to 83)	
	+industry response	322 (220 to 445)		118 (72 to 179)		124 (81 to 184)		103 (66 to 158)	
Race/Ethnicity									
Non-Hispanic White	*consumer behavior*	23<br>(0 to 66)	38<br>(25 to 66)	10<br>(-7 to 42)	8<br>(1 to 23)	18<br>(2 to 52)	13<br>(5 to 30)	22<br>(8 to 52)	9<br>(2 to 21)
	+industry response	63<br>(16 to 139)	59<br>(34 to 104)	32<br>(4 to 79)	20<br>(5 to 44)	41<br>(12 to 95)	24<br>(9 to 48)	44<br>(17 to 97)	19<br>(5 to 37)

© 2021 Du M et al. *JAMA Network Open.*

Non-Hispanic Black	*consumer behavior*	23<br>(-3 to 74)	8<br>(0 to 22)	2<br>(-7 to 24)	4<br>(1 to 10)	10<br>(3 to 24)	3<br>(1 to 7)	6<br>(2 to 16)	2<br>(0 to 5)
	+industry response	52<br>(13 to 126)	17 (<br>5 to 38)	15<br>(-1 to 44)	7<br>(3 to 16)	20<br>(7 to 40)	6<br>(2 to 12)	13<br>(4 to 29)	4<br>(1 to 8)
Hispanic	*consumer behavior*	14<br>(-8 to 56)	19<br>(4 to 49)	2<br>(-6 to 21)	9<br>(4 to 18)	4<br>(0 to 13)	4<br>(0 to 13)	4<br>(0 to 11)	2<br>(1 to 5)
	+industry response	48<br>(4 to 119)	42<br>(10 to 93)	13<br>(-1 to 41)	16<br>(6 to 32)	11<br>(2 to 27)	8<br>(2 to 21)	9<br>(2 to 22)	5<br>(2 to 10)
Other	*consumer behavior*	7<br>(2 to 17)	5<br>(1 to 12)	3<br>(-2 to 10)	1<br>(0 to 4)	2<br>(0 to 7)	2<br>(1 to 4)	2<br>(0 to 5)	1<br>(0 to 2)
	+industry response	15<br>(6 to 31)	8<br>(3 to 18)	5<br>(1 to 14)	3<br>(1 to 5)	4<br>(1 to 11)	3<br>(2 to 5)	3<br>(1 to 8)	2<br>(1 to 3)
**Advanced Prostate**									
Age	*consumer behavior*	190 (125 to 325)		63 (24 to 138)		70 (34 to 144)		50 (19 to 112)	
	+industry response	330 (201 to 539)		135 (64 to 257)		133 (65 to 240)		105 (39 to 200)	
Race/Ethnicity									
Non-Hispanic White	*consumer behavior*	0	130<br>(80 to 239)	0	32<br>(1 to 104)	0	50<br>(17 to 122)	0	37<br>(7 to 99)
	+industry response	0	204<br>(103 to 381)	0	79<br>(18 to 201)	0	95<br>(31 to 201)	0	78<br>(19 to 177)
Non-Hispanic Black	*consumer behavior*	0	26<br>(1 to 87)	0	15<br>(3 to 41)	0	11<br>(4 to 26)	0	8<br>(3 to 19)
	+industry response	0	59<br>(14 to 145)	0	28<br>(10 to 67)	0	22<br>(8 to 48)	0	15<br>(6 to 31)
Hispanic	*consumer behavior*	0	20<br>(4 to 52)	0	10<br>(4 to 21)	0	4<br>(-1 to 15)	0	3<br>(1 to 8)
	+industry response	0	44<br>(11 to 99)	0	18<br>(7 to 36)	0	9<br>(2 to 24)	0	6<br>(2 to 14)
Other	*consumer behavior*	0	6<br>(1 to 18)	0	3<br>(0 to 6)	0	3<br>(1 to 7)	0	1<br>(0 to 4)
	+industry response	0	12<br>(5 to 27)	0	4<br>(2 to 9)	0	4<br>(2 to 9)	0	3<br>(1 to 6)
**Ovarian**									
Age	*consumer behavior*	69 (4 to 174)		5 (-22 to 69)		33 (7 to 90)		34 (11 to 90)	
	+industry response	175 (40 to 373)		47 (-7 to 154)		72 (17 to 166)		73 (22 to 165)	
Race/Ethnicity									

© 2021 Du M et al. *JAMA Network Open.*

Non-Hispanic White	*consumer behavior*	38 (-14 to 138)	0	6 (-17 to 68)	0	22 (-2 to 77)	0	28 (7 to 84)	0
	+industry response	98 (-20 to 275)	0	34 (-14 to 134)	0	51 (0 to 142)	0	59 (10 to 149)	0
Non-Hispanic Black	*consumer behavior*	9 (-3 to 40)	0	-2 (-7 to 10)	0	5 (1 to 15)	0	3 (0 to 8)	0
	+industry response	21 (-4 to 71)	0	3 (-7 to 22)	0	9 (1 to 25)	0	6 (1 to 16)	0
Hispanic	*consumer behavior*	7 (-12 to 48)	0	-3 (-9 to 12)	0	3 (0 to 10)	0	2 (-1 to 8)	0
	+industry response	30 (-15 to 107)	0	3 (-9 to 27)	0	7 (0 to 19)	0	5 (-1 to 16)	0
Other	*consumer behavior*	7 (1 to 19)	0	2 (-2 to 10)	0	2 (0 to 6)	0	1 (0 to 4)	0
	+industry response	15 (1 to 36)	0	4 (0 to 14)	0	3 (1 to 9)	0	2 (0 to 6)	0

[a] Values are the median estimates (95% uncertainty intervals) of each distribution of 1000 simulations.

© 2021 Du M et al. *JAMA Network Open.*

eTable 15. Estimated cancer deaths reduced by Nutrition Facts added sugar labeling policy in the US by age, gender, race/ethnicity, and cancer type over a lifetime (US population=235,162,844)[a]

Cancer Type	Policy Scenario	20-44 y		45-54 y		55-64 y		65 + y	
		Female	Male	Female	Male	Female	Male	Female	Male
**Breast (Postmenopausal)**									
Age	*consumer behavior*	2410 (842 to 4700)		160 (-115 to 560)		275 (111 to 552)		205 (91 to 387)	
	+industry response	5990 (3180 to 9790)		625 (236 to 1230)		610 (314 to 1000)		432 (221 to 737)	
Race/Ethnicity									
Non-Hispanic White	*consumer behavior*	1430 (142 to 3590)	0	93 (-115 to 452)	0	164 (18 to 433)	0	146 (51 to 333)	0
	+industry response	3610 (1140 to 7310)	0	369 (29 to 884)	0	389 (126 to 760)	0	307 (113 to 606)	0
Non-Hispanic Black	*consumer behavior*	434 (-61 to 1310)	0	35 (-54 to 201)	0	68 (16 to 157)	0	33 (8 to 84)	0
	+industry response	1010 (272 to 2290)	0	152 (5 to 392)	0	131 (49 to 265)	0	72 (22 to 152)	0
Hispanic	*consumer behavior*	249 (-136 to 874)	0	-4 (-55 to 101)	0	22 (2 to 58)	0	13 (-1 to 40)	0
	+industry response	855 (77 to 1880)	0	60 (-24 to 209)	0	54 (13 to 113)	0	35 (8 to 76)	0
Other	*consumer behavior*	186 (61 to 402)	0	12 (-14 to 57)	0	11 (-2 to 34)	0	6 (-1 to 18)	0
	+industry response	389 (150 to 710)	0	25 (1 to 76)	0	21 (7 to 48)	0	12 (4 to 28)	0
**Liver**									
Age	*consumer behavior*	2660 (1830 to 3980)		750 (336 to 1350)		606 (356 to 1010)		388 (228 to 659)	
	+industry response	5040 (3640 to 7300)		1720 (988 to 2680)		1180 (778 to 1780)		782 (468 to 1240)	
Race/Ethnicity									
Non-Hispanic White	*consumer behavior*	193 (25 to 550)	1430 (889 to 2630)	57 (-50 to 253)	275 (3 to 816)	86 (7 to 255)	264 (94 to 610)	89 (31 to 219)	157 (44 to 374)
	+industry response	482 (138 to 1100)	2260 (1200 to 4270)	196 (18 to 493)	673 (143 to 1550)	201 (63 to 452)	493 (189 to 1020)	184 (60 to 402)	322 (89 to 680)
Non-Hispanic Black	*consumer behavior*	105 (-13 to 342)	264 (14 to 761)	19 (-24 to 107)	132 (22 to 360)	28 (5 to 76)	78 (27 to 170)	17 (5 to 45)	41 (14 to 89)

© 2021 Du M et al. *JAMA Network Open.*

	+industry response	243 (54 to 577)	575 (149 to 1260)	74 (-1 to 195)	252 (94 to 575)	57 (20 to 126)	152 (52 to 300)	36 (11 to 82)	75 (30 to 147)
Hispanic	*consumer behavior*	73 (-61 to 301)	360 (86 to 857)	1 (-50 to 118)	160 (68 to 334)	25 (0 to 81)	48 (-11 to 156)	19 (-2 to 58)	24 (4 to 64)
	+industry response	273 (-11 to 667)	797 (198 to 1630)	69 (-19 to 243)	289 (111 to 586)	63 (11 to 149)	107 (22 to 261)	48 (9 to 112)	52 (15 to 114)
Other	*consumer behavior*	29 (9 to 71)	86 (1 to 259)	12 (-9 to 47)	30 (1 to 71)	10 (-2 to 31)	30 (10 to 63)	7 (-1 to 23)	14 (1 to 35)
	+industry response	62 (23 to 130)	158 (56 to 383)	23 (4 to 66)	51 (20 to 104)	20 (6 to 46)	43 (22 to 85)	14 (4 to 35)	23 (9 to 50)
**Endometrial**									
Age	*consumer behavior*	1090 (410 to 2030)		205 (-189 to 735)		394 (161 to 764)		357 (174 to 657)	
	+industry response	2670 (1570 to 4060)		852 (311 to 1640)		860 (445 to 1410)		754 (402 to 1210)	
Race/Ethnicity									
Non-Hispanic White	*consumer behavior*	489 (75 to 1200)	0	157 (-142 to 593)	0	209 (23 to 550)	0	242 (85 to 528)	0
	+industry response	1230 (438 to 2320)	0	540 (52 to 1180)	0	493 (158 to 988)	0	500 (183 to 953)	0
Non-Hispanic Black	*consumer behavior*	333 (-48 to 1000)	0	3 (-129 to 233)	0	118 (24 to 274)	0	66 (18 to 161)	0
	+industry response	776 (209 to 1690)	0	174 (-40 to 488)	0	234 (84 to 446)	0	147 (48 to 283)	0
Hispanic	*consumer behavior*	140 (-63 to 466)	0	-1 (-74 to 143)	0	34 (2 to 90)	0	25 (-1 to 72)	0
	+industry response	458 (44 to 1000)	0	88 (-28 to 284)	0	84 (21 to 173)	0	65 (14 to 139)	0
Other	*consumer behavior*	63 (20 to 143)	0	14 (-18 to 68)	0	14 (-2 to 40)	0	9 (-1 to 29)	0
	+industry response	130 (50 to 247)	0	32 (1 to 95)	0	26 (8 to 58)	0	19 (6 to 44)	0
**Kidney (Renal Cell)**									
Age	*consumer behavior*	1100 (743 to 1610)		342 (136 to 620)		365 (202 to 617)		278 (158 to 465)	
	+industry response	2060 (1480 to 2870)		820 (499 to 1230)		733 (475 to 1070)		570 (334 to 854)	
Race/Ethnicity									
Non-Hispanic White	*consumer behavior*	63 (10 to 156)	589 (369 to 1020)	37 (-38 to 150)	151 (7 to 390)	73 (8 to 197)	169 (51 to 396)	87 (31 to 190)	118 (29 to 282)

© 2021 Du M et al. *JAMA Network Open.*

	+industry response	158 (54 to 319)	938 (523 to 1560)	134 (11 to 293)	364 (102 to 723)	171 (54 to 341)	335 (121 to 643)	184 (67 to 342)	248 (70 to 500)
Non-Hispanic Black	*consumer behavior*	55 (-8 to 172)	106 (4 to 281)	2 (-21 to 43)	43 (10 to 108)	21 (4 to 50)	28 (11 to 57)	13 (3 to 33)	15 (4 to 31)
	+industry response	127 (33 to 300)	230 (69 to 496)	30 (-6 to 88)	81 (33 to 171)	41 (15 to 83)	55 (21 to 96)	30 (9 to 59)	27 (11 to 51)
Hispanic	*consumer behavior*	48 (-27 to 158)	137 (29 to 312)	-1 (-21 to 37)	68 (24 to 140)	13 (1 to 34)	24 (-6 to 73)	10 (0 to 27)	14 (4 to 31)
	+industry response	164 (14 to 345)	307 (72 to 586)	24 (-7 to 75)	131 (48 to 234)	32 (8 to 67)	54 (12 to 119)	25 (5 to 53)	28 (10 to 52)
Other	*consumer behavior*	15 (5 to 35)	35 (3 to 96)	4 (-4 to 18)	11 (1 to 24)	4 (-1 to 11)	12 (4 to 25)	3 (0 to 9)	5 (0 to 13)
	+industry response	32 (12 to 63)	63 (24 to 140)	8 (1 to 25)	19 (7 to 35)	7 (2 to 17)	18 (9 to 34)	6 (2 to 14)	8 (3 to 18)
**Pancreatic**									
Age	*consumer behavior*	603 (350 to 927)		187 (56 to 377)		259 (133 to 443)		217 (122 to 371)	
	+industry response	1240 (816 to 1770)		495 (274 to 794)		527 (330 to 815)		444 (257 to 688)	
Race/Ethnicity									
Non-Hispanic White	*consumer behavior*	100 (8 to 276)	268 (134 to 557)	26 (-44 to 137)	85 (9 to 246)	69 (5 to 203)	104 (27 to 258)	86 (33 to 198)	77 (19 to 183)
	+industry response	258 (76 to 557)	480 (231 to 930)	111 (-2 to 282)	204 (57 to 446)	164 (40 to 357)	208 (68 to 430)	177 (67 to 364)	163 (46 to 327)
Non-Hispanic Black	*consumer behavior*	38 (-5 to 121)	34 (1 to 95)	-3 (-22 to 39)	18 (4 to 48)	21 (5 to 49)	15 (6 to 31)	13 (3 to 35)	8 (2 to 18)
	+industry response	90 (22 to 207)	75 (21 to 168)	23 (-9 to 83)	34 (13 to 75)	40 (15 to 83)	28 (11 to 54)	29 (9 to 64)	16 (6 to 29)
Hispanic	*consumer behavior*	26 (-11 to 92)	60 (13 to 143)	5 (-11 to 40)	23 (7 to 53)	8 (0 to 25)	11 (-2 to 33)	7 (-1 to 22)	7 (2 to 15)
	+industry response	84 (6 to 200)	133 (32 to 279)	25 (0 to 78)	46 (16 to 92)	23 (5 to 52)	24 (5 to 54)	19 (4 to 41)	14 (5 to 27)
Other	*consumer behavior*	13 (4 to 30)	16 (-1 to 53)	5 (-4 to 19)	7 (1 to 17)	5 (-1 to 17)	8 (3 to 16)	4 (-1 to 11)	4 (0 to 9)
	+industry response	26 (9 to 53)	31 (11 to 75)	10 (1 to 27)	12 (5 to 25)	10 (3 to 24)	11 (6 to 22)	8 (2 to 18)	6 (2 to 13)
**Esophageal Adenocarcinoma**									
Age	*consumer behavior*	782 (526 to 1250)		195 (61 to 419)		217 (98 to 437)		157 (72 to 309)	

© 2021 Du M et al. *JAMA Network Open.*

	+industry response	1370 (900 to 2070)		459 (215 to 802)		427 (231 to 741)		326 (152 to 556)	
Race/Ethnicity									
Non-Hispanic White	*consumer behavior*	45 (4 to 114)	616 (373 to 1070)	13 (-13 to 55)	136 (11 to 357)	24 (2 to 62)	158 (49 to 380)	26 (8 to 59)	112 (31 to 260)
	+industry response	116 (36 to 224)	992 (543 to 1660)	46 (5 to 105)	325 (99 to 655)	55 (17 to 112)	312 (119 to 619)	55 (19 to 105)	233 (67 to 461)
Non-Hispanic Black	*consumer behavior*	7 (-1 to 21)	14 (1 to 36)	0 (-3 to 6)	7 (2 to 17)	3 (1 to 7)	5 (2 to 9)	2 (0 to 5)	3 (1 to 6)
	+industry response	16 (4 to 35)	29 (9 to 61)	4 (-1 to 12)	13 (5 to 27)	6 (2 to 12)	9 (4 to 16)	4 (1 to 9)	5 (2 to 10)
Hispanic	*consumer behavior*	12 (-5 to 38)	63 (14 to 143)	0 (-4 to 9)	28 (11 to 56)	3 (0 to 8)	11 (-2 to 32)	2 (0 to 6)	6 (1 to 13)
	+industry response	38 (4 to 80)	137 (34 to 264)	3 (-1 to 18)	51 (20 to 93)	7 (2 to 15)	23 (6 to 53)	5 (1 to 12)	12 (4 to 24)
Other	*consumer behavior*	5 (2 to 12)	11 (1 to 29)	2 (-1 to 7)	4 (0 to 9)	2 (0 to 5)	4 (1 to 8)	1 (0 to 3)	2 (0 to 5)
	+industry response	11 (4 to 20)	19 (7 to 41)	3 (0 to 9)	6 (3 to 12)	3 (1 to 7)	6 (3 to 11)	2 (1 to 5)	3 (1 to 7)
**Colorectal**									
Age	*consumer behavior*	464 (314 to 674)		92.6 (33 to 172)		121 (67 to 203)		100 (56 to 168)	
	+industry response	872 (604 to 1220)		231 (132 to 360)		239 (152 to 375)		203 (119 to 315)	
Race/Ethnicity									
Non-Hispanic White	*consumer behavior*	64 (6 to 189)	234 (150 to 399)	12 (-17 to 69)	35 (0 to 100)	31 (2 to 91)	47 (17 to 113)	42 (15 to 94)	31 (8 to 75)
	+industry response	161 (43 to 377)	367 (196 to 623)	50 (0 to 134)	85 (20 to 195)	74 (19 to 165)	89 (34 to 189)	88 (32 to 176)	65 (18 to 141)
Non-Hispanic Black	*consumer behavior*	19 (-4 to 68)	25 (1 to 73)	-2 (-10 to 15)	11 (2 to 30)	9 (2 to 22)	9 (3 to 19)	5 (1 to 16)	5 (1 to 12)
	+industry response	47 (9 to 125)	54 (13 to 124)	9 (-6 to 35)	21 (7 to 47)	17 (5 to 36)	16 (6 to 33)	12 (3 to 29)	10 (4 to 20)
Hispanic	*consumer behavior*	21 (-3 to 71)	45 (8 to 114)	1 (-6 to 19)	18 (7 to 37)	4 (0 to 13)	6 (-1 to 20)	3 (0 to 10)	4 (1 to 10)
	+industry response	59 (9 to 144)	100 (21 to 218)	11 (-2 to 36)	32 (12 to 64)	10 (2 to 24)	14 (3 to 36)	8 (1 to 21)	9 (3 to 18)
Other	*consumer behavior*	9 (3 to 21)	17 (0 to 48)	2 (-2 to 9)	4 (0 to 10)	2 (0 to 7)	4 (1 to 9)	2 (0 to 5)	2 (0 to 5)
	+industry response	18 (6 to 37)	32 (12 to 71)	5 (0 to 13)	8 (3 to 15)	4 (1 to 10)	6 (3 to 12)	3 (1 to 8)	3 (1 to 7)

© 2021 Du M et al. *JAMA Network Open.*

**Stomach (Gastric Cardia)**									
Age	*consumer behavior*	310 (199 to 566)		74 (19 to 202)		89 (38 to 206)		68 (31 to 144)	
	+industry response	553 (328 to 973)		191 (74 to 393)		182 (83 to 356)		138 (62 to 265)	
Race/Ethnicity									
Non-Hispanic White	*consumer behavior*	20 (-2 to 70)	236 (141 to 473)	1 (-11 to 29)	54 (5 to 182)	12 (1 to 39)	58 (12 to 169)	14 (5 to 34)	43 (10 to 122)
	+industry response	54 (7 to 137)	379 (181 to 764)	17 (-7 to 63)	134 (27 to 331)	28 (5 to 72)	121 (31 to 299)	29 (9 to 63)	89 (19 to 220)
Non-Hispanic Black	*consumer behavior*	4 (-1 to 17)	4 (0 to 21)	0 (-2 to 4)	4 (1 to 12)	2 (1 to 4)	3 (1 to 7)	1 (0 to 3)	1 (0 to 4)
	+industry response	10 (1 to 30)	9 (0 to 39)	2 (-1 to 9)	8 (2 to 20)	3 (1 to 7)	6 (2 to 13)	2 (1 to 5)	3 (1 to 7)
Hispanic	*consumer behavior*	2 (-8 to 24)	23 (3 to 64)	-1 (-4 to 7)	8 (2 to 20)	1 (0 to 5)	3 (-1 to 12)	1 (0 to 4)	2 (1 to 6)
	+industry response	17 (-5 to 56)	52 (9 to 119)	3 (-3 to 15)	16 (3 to 36)	4 (0 to 9)	8 (1 to 22)	3 (0 to 7)	5 (1 to 11)
Other	*consumer behavior*	2 (0 to 7)	6 (0 to 19)	1 (-1 to 5)	2 (-1 to 6)	1 (0 to 4)	3 (1 to 7)	1 (0 to 2)	1 (0 to 4)
	+industry response	5 (1 to 14)	11 (3 to 29)	2 (0 to 7)	4 (1 to 10)	2 (1 to 5)	5 (3 to 9)	1 (0 to 4)	2 (1 to 5)
**Multiple Myeloma**									
Age	*consumer behavior*	164 (82 to 292)		60 (18 to 130)		90 (53 to 158)		69 (35 to 124)	
	+industry response	364 (200 to 584)		158 (81 to 275)		173 (105 to 291)		140 (72 to 239)	
Race/Ethnicity									
Non-Hispanic White	*consumer behavior*	16 (-2 to 63)	57 (16 to 160)	10 (-6 to 48)	23 (0 to 82)	16 (0 to 57)	38 (17 to 91)	22 (6 to 60)	22 (3 to 70)
	+industry response	43 (1 to 125)	116 (29 to 295)	31 (-3 to 94)	57 (8 to 154)	38 (5 to 103)	67 (25 to 147)	44 (11 to 109)	49 (8 to 124)
Non-Hispanic Black	*consumer behavior*	15 (-2 to 64)	22 (0 to 76)	-3 (-13 to 24)	11 (3 to 32)	11 (4 to 32)	7 (1 to 21)	7 (1 to 21)	6 (2 to 14)
	+industry response	34 (3 to 117)	48 (7 to 135)	10 (-11 to 49)	21 (7 to 53)	22 (6 to 54)	15 (3 to 39)	15 (2 to 40)	10 (4 to 24)
Hispanic	*consumer behavior*	2 (-12 to 31)	24 (4 to 74)	-3 (-8 to 10)	8 (2 to 23)	3 (-0 to 9)	4 (-1 to 16)	2 (-1 to 9)	3 (1 to 7)
	+industry response	21 (-10 to 79)	54 (7 to 140)	3 (-6 to 25)	17 (4 to 43)	7 (0 to 18)	9 (1 to 27)	6 (-0 to 17)	5 (1 to 13)

© 2021 Du M et al. *JAMA Network Open.*

Other	*consumer behavior*	2 (0 to 6)	7 (1 to 20)	1 (-1 to 4)	2 (0 to 6)	1 (0 to 3)	3 (1 to 6)	1 (0 to 2)	1 (0 to 3)
	+industry response	4 (1 to 12)	12 (4 to 30)	2 (0 to 6)	4 (1 to 9)	2 (1 to 5)	4 (2 to 8)	1 (0 to 4)	2 (1 to 4)
**Gallbladder**									
Age	*consumer behavior*	126 (74 to 192)		39 (13 to 72)		53 (31 to 84)		41 (24 to 67)	
	+industry response	275 (187 to 384)		101 (61 to 156)		106 (69 to 155)		84 (53 to 127)	
Race/Ethnicity									
Non-Hispanic White	*consumer behavior*	22 (0 to 63)	32 (21 to 55)	9 (-6 to 37)	7 (1 to 19)	15 (1 to 44)	11 (4 to 25)	18 (6 to 42)	7 (2 to 17)
	+industry response	58 (16 to 130)	50 (27 to 87)	28 (3 to 71)	16 (4 to 36)	35 (10 to 79)	20 (8 to 41)	36 (14 to 79)	15 (4 to 30)
Non-Hispanic Black	*consumer behavior*	20 (-2 to 64)	6 (0 to 18)	2 (-6 to 21)	3 (0 to 7)	9 (2 to 21)	2 (1 to 5)	5 (1 to 12)	1 (0 to 3)
	+industry response	46 (12 to 110)	13 (4 to 30)	13 (-1 to 40)	6 (2 to 12)	17 (6 to 35)	5 (2 to 10)	10 (3 to 22)	3 (1 to 6)
Hispanic	*consumer behavior*	13 (-7 to 48)	15 (3 to 38)	2 (-5 to 18)	8 (3 to 16)	4 (-0 to 12)	3 (0 to 11)	3 (0 to 10)	2 (1 to 4)
	+industry response	42 (3 to 101)	33 (8 to 74)	12 (-1 to 36)	14 (6 to 28)	10 (2 to 23)	7 (2 to 18)	8 (2 to 19)	4 (1 to 8)
Other	*consumer behavior*	6 (2 to 15)	4 (0 to 10)	2 (-2 to 9)	1 (0 to 2)	2 (0 to 6)	2 (1 to 4)	1 (0 to 4)	1 (0 to 2)
	+industry response	13 (5 to 26)	6 (2 to 14)	4 (1 to 12)	1 (0 to 3)	4 (1 to 9)	3 (1 to 5)	3 (1 to 7)	1 (0 to 3)
**Advanced Prostate**									
Age	*consumer behavior*	103 (64 to 179)		26 (11 to 54)		21 (10 to 41)		21 (8 to 45)	
	+industry response	178 (108 to 302)		54 (27 to 100)		40 (21 to 70)		44 (17 to 82)	
Race/Ethnicity									
Non-Hispanic White	*consumer behavior*	0	67 (38 to 134)	0	11 (0 to 37)	0	13 (4 to 32)	0	14 (3 to 39)
	+industry response	0	106 (54 to 214)	0	28 (6 to 71)	0	25 (8 to 52)	0	31 (8 to 70)
Non-Hispanic Black	*consumer behavior*	0	13 (1 to 44)	0	6 (1 to 18)	0	4 (2 to 10)	0	4 (1 to 10)
	+industry response	0	28 (6 to 80)	0	12 (4 to 28)	0	9 (3 to 20)	0	8 (3 to 16)

© 2021 Du M et al. *JAMA Network Open.*

Hispanic	*consumer behavior*	0	16 (3 to 41)	0	6 (2 to 12)	0	2 (-1 to 6)	0	1 (0 to 4)
	+industry response	0	34 (8 to 78)	0	10 (4 to 21)	0	4 (1 to 11)	0	3 (1 to 7)
Other	*consumer behavior*	0	2 (0 to 9)	0	1 (0 to 2)	0	1 (0 to 2)	0	1 (0 to 2)
	+industry response	0	4 (0 to 14)	0	1 (1 to 3)	0	1 (1 to 2)	0	1 (0 to 2)
**Ovarian**									
Age	*consumer behavior*	47 (9 to 110)		4 (-16 to 49)		25 (6 to 65)		26 (8 to 67)	
	+industry response	118 (36 to 236)		36 (-2 to 112)		55 (16 to 120)		56 (17 to 125)	
Race/Ethnicity									
Non-Hispanic White	*consumer behavior*	25 (-4 to 82)	0	5 (-11 to 50)	0	16 (-1 to 56)	0	21 (5 to 62)	0
	+industry response	64 (-3 to 167)	0	26 (-8 to 96)	0	38 (2 to 102)	0	45 (9 to 111)	0
Non-Hispanic Black	*consumer behavior*	6 (-2 to 28)	0	-2 (-5 to 8)	0	4 (1 to 12)	0	2 (0 to 7)	0
	+industry response	15 (-1 to 48)	0	3 (-5 to 18)	0	7 (2 to 20)	0	5 (1 to 13)	0
Hispanic	*consumer behavior*	6 (-7 to 35)	0	-2 (-6 to 9)	0	2 (0 to 8)	0	1 (-1 to 7)	0
	+industry response	22 (-7 to 76)	0	2 (-6 to 20)	0	6 (0 to 15)	0	4 (-1 to 13)	0
Other	*consumer behavior*	5 (1 to 13)	0	1 (-2 to 7)	0	1 (0 to 4)	0	1 (0 to 3)	0
	+industry response	10 (1 to 25)	0	3 (0 to 10)	0	2 (1 to 6)	0	1 (0 to 5)	0
			0						
**Thyroid**									
Age	*consumer behavior*	8 (3 to 16)		4 (1 to 11)		5 (3 to 10)		7 (3 to 11)	
	+industry response	17 (7 to 32)		11 (4 to 22)		10 (6 to 17)		13 (8 to 22)	
Race/Ethnicity									
Non-Hispanic White	*consumer behavior*	0 (0 to 3)	1 (0 to 7)	0 (0 to 2)	2 (0 to 7)	0 (0 to 2)	2 (1 to 6)	2 (0 to 6)	1 (0 to 4)
	+industry response	0 (0 to 6)	2 (0 to 11)	1 (0 to 4)	5 (1 to 14)	1 (0 to 3)	4 (1 to 11)	4 (1 to 11)	3 (1 to 7)

© 2021 Du M et al. *JAMA Network Open.*

Non-Hispanic Black	*consumer behavior*	1 (0 to 5)	1 (0 to 5)	0 (0 to 1)	0 (0 to 1)	0 (0 to 2)	0 (0 to 1)	1 (0 to 2)	0 (0 to 1)
	+industry response	2 (0 to 10)	3 (0 to 9)	0 (0 to 3)	0 (0 to 2)	1 (0 to 3)	0 (0 to 1)	1 (0 to 4)	0 (0 to 1)
Hispanic	*consumer behavior*	1 (0 to 7)	0 (0 to 3)	0 (-1 to 2)	1 (0 to 3)	0 (0 to 1)	0 (0 to 2)	1 (0 to 3)	0 (0 to 1)
	+industry response	5 (0 to 16)	1 (0 to 6)	1 (-1 to 5)	2 (0 to 5)	1 (0 to 3)	1 (0 to 3)	2 (0 to 5)	1 (0 to 2)
Other	*consumer behavior*	0	0 (0 to 2)	0 (0 to 1)	0 (0 to 1)	0	0	0 (0 to 1)	0 (0 to 1)
	+industry response	0	1 (0 to 4)	0 (0 to 1)	1 (0 to 2)	0 (0 to 1)	0 (0 to 1)	1 (0 to 2)	0 (0 to 1)

[a] Values are the median estimates (95% uncertainty intervals) of each distribution of 1000 simulations.

© 2021 Du M et al. *JAMA Network Open.*

eTable 16. Estimated health gains and costs associated with Nutrition Facts added sugar labeling on reducing cancer burdens in the US over 10 years (US population=235,162,844)[a]

	**Added Sugar Labeling Policy**	
	**Consumer Behavior** Median (2.5% to 97.5%)	**Consumer Behavior + Industry Response** Median (2.5% to 97.5%)
**New Cancer Cases Averted, N (95% UI)**		
Endometrial cancer	1200 (756 to 1690)	2470 (1730 to 3230)
Breast cancer (postmenopausal)	1220 (743 to 1770)	2510 (1690 to 3360)
Kidney cancer	690 (472 to 955)	1510 (1160 to 1910)
Liver cancer	471 (321 to 662)	1010 (761 to 1310)
Esophageal adenocarcinoma	163 (82 to 270)	378 (239 to 536)
Pancreatic cancer	212 (137 to 297)	460 (335 to 593)
Colorectal cancer	231 (154 to 318)	513 (386 to 659)
Stomach cancer (cardia)	63 (29 to 114)	153 (95 to 237)
Multiple myeloma	80 (46 to 125)	190 (133 to 260)
Thyroid cancer	123 (83 to 175)	268 (195 to 355)
Advanced prostate cancer	52 (22 to 96)	129 (76 to 194)
Gallbladder cancer	41 (28 to 57)	89 (67 to 113)
Ovarian cancer	52 (31 to 82)	107 (66 to 155)
Total	4650 (3170 to 6250)	9980 (7560 to 12300)
**Cancer Deaths Prevented, N (95% UI)**		
Liver cancer	215 (143 to 307)	468 (353 to 612)
Breast cancer (postmenopausal)	92 (62 to 128)	195 (142 to 252)
Endometrial cancer	101 (63 to 142)	211 (154 to 275)
Kidney cancer	75 (47 to 110)	173 (129 to 230)
Esophageal adenocarcinoma	66 (31 to 114)	162 (102 to 231)
Pancreatic cancer	111 (69 to 158)	248 (176 to 327)
Colorectal cancer	41 (26 to 57)	93 (69 to 121)
Stomach cancer (cardia)	23 (9 to 46)	61 (36 to 98)
Multiple myeloma	16 (8 to 28)	42 (27 to 63)
Gallbladder cancer	19 (13 to 27)	41 (31 to 53)
Advanced prostate cancer	4 (0 to 9)	13 (7 to 21)
Ovarian cancer	15 (9 to 25)	31 (19 to 47)
Thyroid cancer	1 (0 to 2)	3 (2 to 4)
Total	794 (529 to 1060)	1780 (1350 to 2230)
**Life Years Gained**	841 (548 to 1160)	1910 (1430 to 2410)
**QALYs Gained**	5890 (3900 to 8030)	12900 (9510 to 16400)
**Changes in Health-Related Costs, Cancer Only** ($, millions)[b,c]		
Medical cost	-250 (-332 to – 172)	-542 (-664 to -421)
Patient time cost	-10.9 (-15.4 to -7.13)	-24.0 (-30.5 to -17.8)
Productivity loss	-67.5 (-91.6 to -44.8)	-148 (-183 to -112)
**Policy Implementation Costs** ($, millions)[b,c]		
Government cost	6.55 (5.68 to 7.87)	6.56 (5.74 to 7.91)
Administration	4.53 (4.30 to 4.79)	4.53 (4.31 to 4.79)
Monitoring	2.00 (1.19 to 3.34)	2.01 (1.25 to 3.37)
Industry cost	1660 (1390 to 1950)	2040 (1740 to 2330)
Compliance	1660 (1390 to 1950)	1660 (1390 to 1950)
Reformulation	-------	367 (300 to 443)

© 2021 Du M et al. *JAMA Network Open.*

**Net Costs, Cancer Only** ($, millions) [b,c,d]		
Societal perspective	1340 (1070 to 1650)	1330 (992 to 1660)
Healthcare perspective	-243 (-325 to -165)	-535 (-657 to -414)
**ICER** (dollars/QALY)[e]		
Societal perspective	228000 (148000 to 381000)	105100 (66827 to 167000)
Healthcare perspective	Cost-saving	Cost-saving

Abbreviations: ICER, Incremental Cost-Effectiveness Ratio; QALY, quality-adjusted life years.
[a] Values are the median estimates (95% uncertainty intervals) of each distribution of 1000 simulations.
[b] Health-related costs were inflated to 2015 US dollars using the Personal Health Care (PHC) index. Policy intervention costs were inflated to 2015 US dollars using the Consumer Price Index. Negative costs represent savings.
[c] Costs are medians from 1000 simulations so may not add up to totals.
[d] Net costs were calculated as policy costs minus health-related costs from reduced cancer burdens. Societal perspective includes healthcare cost, patient time costs, productivity costs, and policy implementation costs; government perspective included policy costs relevant to policy implementation and program monitoring and evaluation and medical costs.
[e] ICER threshold was evaluated at $150 000/QALY.

© 2021 Du M et al. *JAMA Network Open.*

eTable 17. Estimated health gains and costs associated with Nutrition Facts added sugar labeling on reducing cancer burdens in the US over a lifetime, 1-way sensitivity analyses at 50% of the policy impact and the diet-BMI association (US population=235,162,844)[a]

	Added Sugar Labeling Policy				
	50% Policy Impact			50% diet-BMI Association	
	**Consumer Behavior** Median (2.5% to 97.5%)	**Consumer Behavior + Industry Reformulation** Median (2.5% to 97.5%)		**Consumer Behavior** Median (2.5% to 97.5%)	**Consumer Behavior + Industry Reformulation** Median (2.5% to 97.5%)
**New Cancer Cases Averted, N (95% UI)**					
Endometrial cancer	2270 (71 to 4960)	6960 (4440 to 10300)		3120 (1750 to 4750)	7690 (5470 to 10200)
Breast cancer (postmenopausal)	2230 (266 to 4620)	6460 (4040 to 9670)		2880 (1680 to 4440)	6950 (4990 to 9520)
Kidney cancer	3140 (2130 to 4260)	5930 (4620 to 7300)		2740 (2080 to 3470)	5450 (4350 to 6620)
Liver cancer	2970 (2090 to 4220)	5500 (4290 to 7180)		2540 (1960 to 3360)	4980 (3960 to 6320)
Esophageal adenocarcinoma	976 (628 to 1400)	1710 (1280 to 2250)		792 (572 to 1070)	1510 (1120 to 1960)
Pancreatic cancer	750 (442 to 1100)	1600 (1190 to 2080)		729 (518 to 961)	1550 (1210 to 1920)
Colorectal cancer	692 (471 to 926)	1300 (994 to 1640)		599 (449 to 760)	1190 (937 to 1470)
Stomach cancer (cardia)	405 (252 to 633)	734 (532 to 1050)		339 (237 to 506)	664 (471 to 938)
Multiple myeloma	328 (181 to 518)	715 (494 to 999)		325 (218 to 466)	698 (500 to 934)
Thyroid cancer	297 (143 to 502)	670 (449 to 990)		305 (199 to 462)	669 (472 to 946)
Advanced prostate cancer	241 (161 to 351)	408 (296 to 560)		190 (135 to 262)	351 (254 to 478)
Gallbladder cancer	158 (93 to 231)	344 (266 to 443)		156 (117 to 203)	337 (272 to 418)
Ovarian cancer	57 (-3 to 148)	180 (74 to 318)		79 (32 to 145)	199 (99 to 318)
Total	14800 (3170 to 6250)	32800 (24700 to 42500)		15000 (10800 to 19700)	32500 (25700 to 39900)
**Cancer Deaths Prevented, N (95% UI)**					
Liver cancer	2610 (1820 to 3710)	4810 (3720 to 6290)		2220 (1710 to 2950)	4350 (3440 to 5540)
Breast cancer (postmenopausal)	1160 (-77 to 2920)	3580 (1920 to 6070)		1590 (776 to 2790)	3960 (2470 to 5910)
Endometrial cancer	753 (51 to 1730)	2360 (1510 to 3580)		1050 (617 to 1640)	2620 (1880 to 3500)
Kidney cancer	1190 (788 to 1660)	2260 (1750 to 2850)		1040 (784 to 1340)	2090 (1680 to 2540)
Esophageal adenocarcinoma	846 (545 to 1210)	1470 (1090 to 1940)		680 (494 to 922)	1290 (957 to 1670)
Pancreatic cancer	655 (390 to 961)	1400 (1040 to 1800)		636 (451 to 834)	1350 (1060 to 1680)
Colorectal cancer	452 (306 to 607)	850 (659 to 1080)		393 (296 to 501)	779 (612 to 968)
Stomach cancer (cardia)	334 (206 to 525)	603 (435 to 860)		279 (196 to 417)	543 (384 to 769)
Multiple myeloma	197 (114 to 316)	429 (303 to 591)		194 (133 to 278)	418 (309 to 552)
Gallbladder cancer	130 (76 to 193)	290 (219 to 376)		132 (97 to 172)	286 (230 to 354)
Advanced prostate cancer	108 (71 to 167)	183 (129 to 259)		85 (60 to 123)	157 (112 to 219)
Ovarian cancer	42 (-1 to 102)	130 (62 to 220)		57 (25 to 102)	142 (77 to 215)
Thyroid cancer	13 (6 to 20)	27 (18 to 39)		12 (8 to 18)	26 (18 to 36)
Total	8730 (5080 to 12800)	18600 (14000 to 24100)		8540 (6190 to 11400)	18200 (14300 to 22500)

© 2021 Du M et al. *JAMA Network Open.*

**Life Years Gained**	39400 (22300 to 58500)	85600 (63600 to 111000)		39200 (28200 to 52800)	84100 (66000 to 105000)
**QALYs Gained**	57800 (32100 to 86900)	128000 (95100 to 166000)		58100 (41902 to 76600)	126000 (99500 to 156000)
**Changes in Health-Related Costs, Cancer Only ($, millions)**[b,c]					
Medical cost	-830 (-506 to - 1180)	-1760 (-2200 to -1340)		-800 (-1020 to -596)	-1700 (-2040 to -1360)
Patient time cost	-54.0 (-86.0 to -26.9)	-126 (-164 to -93.0)		-57.0 (-75.7 to -40.3)	-126 (-155 to -100)
Productivity loss	-320 (-501 to -164)	-740 (-970 to -548)		-335 (-445 to -239)	-740 (-909 to -585)
**Policy Implementation Costs ($, millions)**[b,c]					
Government cost	9.24 (7.18 to 12.8)	9.30 (7.28 to 12.5)		9.24 (7.18 to 12.8)	9.30 (7.28 to 12.5)
Administration	4.53 (4.29 to 4.80)	4.53 (4.29 to 4.77)		4.53 (4.29 to 4.80)	4.53 (4.29 to 4.77)
Monitoring	4.69 (2.75 to 8.29)	4.77 (2.69 to 7.96)		4.69 (2.75 to 8.29)	4.77 (2.69 to 7.96)
Industry cost	1660 (1410 to 1960)	2540 (2240 to 2880)		1660 (1410 to 1960)	2540 (2240 to 2880)
Compliance	1660 (1410 to 1960)	1660 (1400 to 1970)		1660 (1410 to 1960)	1660 (1400 to 1970)
Reformulation	-------	869 (718 to 1061)		-------	869 (718 to 1061)
**Net Costs, Cancer Only ($, millions)** [b,c,d]					
Societal perspective	482 (122 to 1020)	-63.2 (-851 to 642)		479 (49.0 to 884)	-16.0 (-662 to 604)
Healthcare perspective	-820 (-1170 to -496)	-1750 (-2190 to -1330)		-790 (-1000 to -586)	-1690 (-2030 to -1350)
**ICER (dollars/QALY)**[e]					
Societal perspective	8330 (-1670 to 30200)	Cost-saving		8320 (709 to 19750)	Cost-saving
Healthcare perspective	Cost-saving	Cost-saving		Cost-saving	Cost-saving

Abbreviations: ICER, Incremental Cost-Effectiveness Ratio; QALY, quality-adjusted life years.
[a] Values are the median estimates (95% uncertainty intervals) of each distribution of 1000 simulations.
[b] Health-related costs were inflated to 2015 US dollars using the Personal Health Care (PHC) index. Policy intervention costs were inflated to 2015 US dollars using the Consumer Price Index. Negative costs represent savings.
[c] Costs are medians from 1000 simulations so may not add up to totals.
[d] Net costs were calculated as policy costs minus health-related costs from reduced cancer burdens. Societal perspective includes healthcare cost, patient time costs, productivity costs, and policy implementation costs; government perspective included policy costs relevant to policy implementation and program monitoring and evaluation and medical costs.
[e] ICER threshold was evaluated at $150 000/QALY.

© 2021 Du M et al. *JAMA Network Open.*

eTable 18. Estimated health gains and costs associated with Nutrition Facts added sugar labeling on reducing cancer burdens in the US over a lifetime, threshold analyses on policy impact (US population=235,162,844)[a]

	**Added Sugar Labeling Policy**			
	**Consumer Behavior (1.65%)** Median (2.5% to 97.5%)	**Consumer Behavior (4.55%)** Median (2.5% to 97.5%)		**Consumer Behavior (3.23%) + Industry Reformulation (4.04%)** Median (2.5% to 97.5%)
**New Cancer Cases Averted, N (95% UI)**	7650 (1230 to 13500)	20900 (14400 to 27700)		32600 (26000 to 39900)
**Cancer Deaths Prevented, N (95% UI)**	4760 (1440 to 7950)	12100 (8510 to 15700)		18500 (14900 to 22500)
**Life Years Gained**	20900 (5240 to 35700)	55000 (38100 to 72200)		85000 (67600 to 104000)
**QALYs Gained**	29900 (5170 to 52900)	81500 (54200 to 109000)		126000 (101000 to 156000)
**Changes in Health-Related Costs, Cancer Only ($, millions)[b,c]**				
Medical cost	-458 (-749 to -158)	-1140 (-1440 to -809)		-1740 (-2100 to -1410)
Patient time cost	-25.0 (-51.5 to 2.28)	-77.7 (-106 to -50.6)		-124 (-155 to -97)
Productivity loss	-149 (-303 to 5.86)	-458 (-620 to -301)		-729 (-902 to -574)
**Policy Implementation Costs ($, millions)[b,c]**				
Government cost	9.24 (7.18 to 12.8)	9.24 (7.18 to 12.8)		9.30 (7.28 to 12.5)
Administration	4.53 (4.29 to 4.80)	4.53 (4.29 to 4.80)		4.53 (4.29 to 4.77)
Monitoring	4.69 (2.75 to 8.29)	4.69 (2.75 to 8.29)		4.77 (2.69 to 7.96)
Industry cost	1660 (1410 to 1960)	1660 (1410 to 1960)		2540 (2240 to 2880)
Compliance	1660 (1410 to 1960)	1660 (1410 to 1960)		1660 (1400 to 1970)
Reformulation	-------	-------		869 (718 to 1060)
**Net Costs, Cancer Only ($, millions)[b,c,d]**				
Societal perspective	1050 (510 to 1540)	10.9 (-566 to 526)		-42.0 (-687 to 591)
Healthcare perspective	-448 (-741 to -148)	-1130 (-1430 to -801)		-1730 (-2090 to -1400)
**ICER (dollars/QALY)[e]**				
Societal perspective	34900 (9190 to 233000)	135 (-5490 to 8870)		Cost-saving
Healthcare perspective	Cost-saving	Cost-saving		Cost-saving

Abbreviations: ICER, Incremental Cost-Effectiveness Ratio; QALY, quality-adjusted life years.
[a] Values are the median estimates (95% uncertainty intervals) of each distribution of 1000 simulations.
[b] Health-related costs were inflated to 2015 US dollars using the Personal Health Care (PHC) index. Policy intervention costs were inflated to 2015 US dollars using the Consumer Price Index. Negative costs represent savings.
[c] Costs are medians from 1000 simulations so may not add up to totals.
[d] Net costs were calculated as policy costs minus health-related costs from reduced cancer burdens. Societal perspective includes healthcare cost, patient time costs, productivity costs, and policy implementation costs; government perspective included policy costs relevant to policy implementation and program monitoring and evaluation and medical costs.

© 2021 Du M et al. *JAMA Network Open.*

[e] ICER threshold was evaluated at $150 000/QALY.

© 2021 Du M et al. *JAMA Network Open.*

eFigure 1. Diet and Cancer Outcome Model (DiCOM)

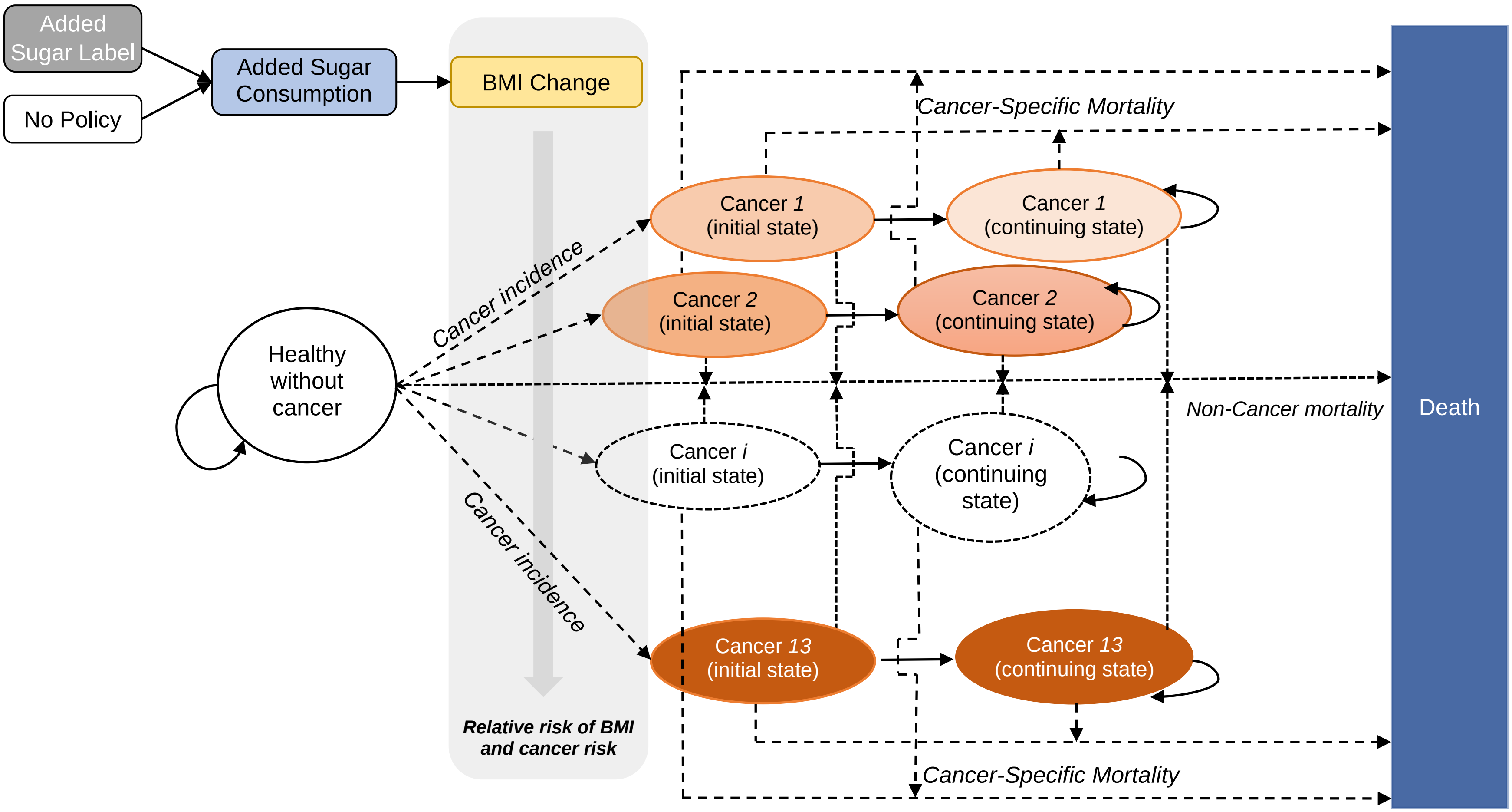

The model consists of four general health states: (a) healthy without cancer (healthy state); (b) initial cancer diagnosis (initial state) for each cancer type *i*; (c) continuing care (continuing state) for each cancer type *i*; and (d) death state. Transitions between states are based on national cancer incidence and cancer-specific mortality rates from SEER (for individual with cancer) and lifetable-based mortality rates (for individuals without cancer). The model simulates the policy impact on the number of new cases and deaths of 13 obesity-associated cancers, health-related quality of life (HRQOL), and health-related costs among U.S. adults over a lifetime by comparing a policy scenario (added sugar label) to a non-policy scenario (status quo).

© 2021 Du M et al. *JAMA Network Open.*

eFigure 2. Estimated reduced new cancer cases and deaths associated with Nutrition Facts added sugar labeling policy in the US by age, gender, race/ethnicity, and cancer type over a lifetime

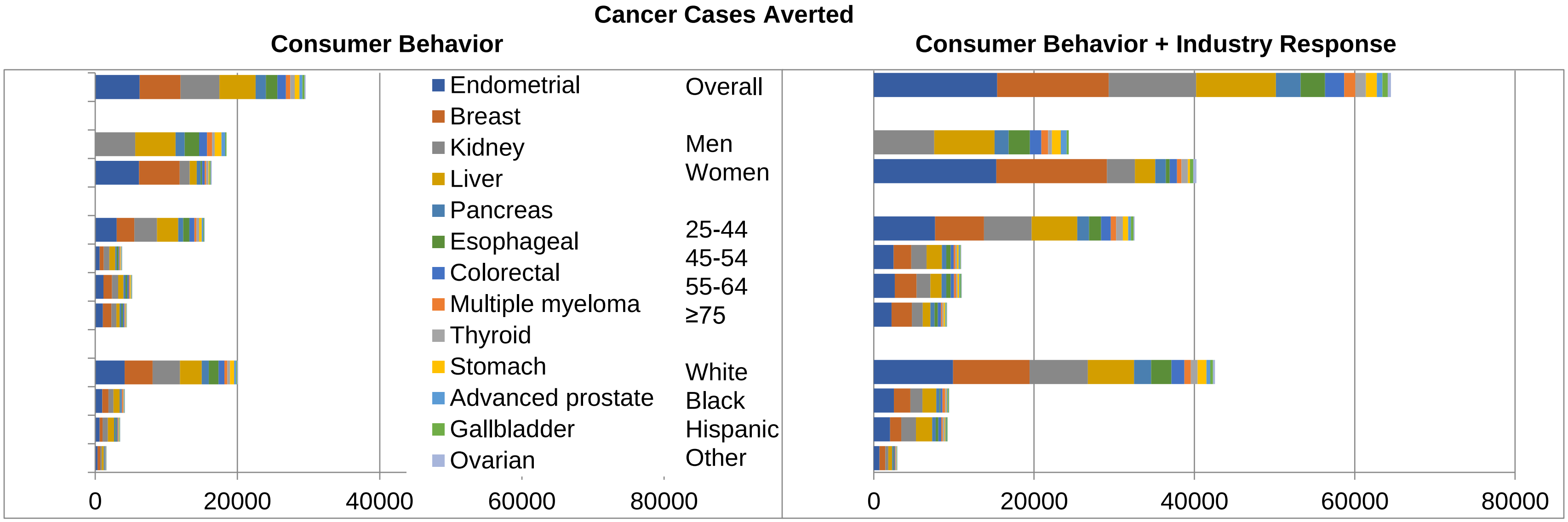

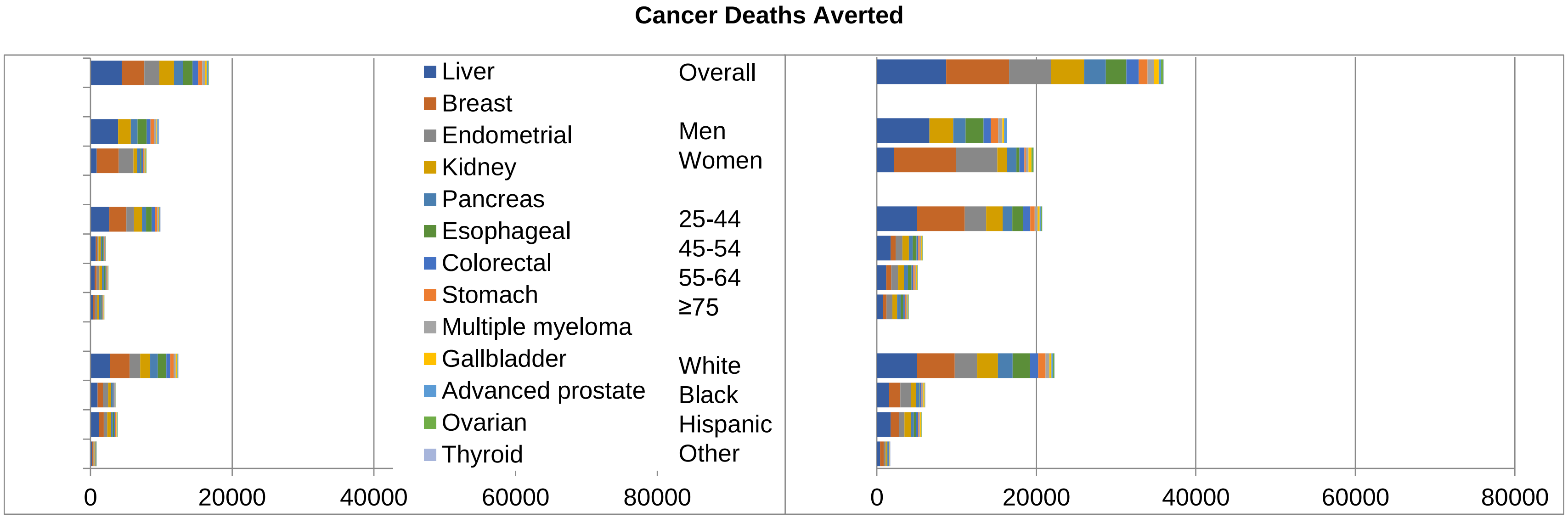

© 2021 Du M et al. *JAMA Network Open.*

eFigure 3. Estimated life years and QALYs gained associated with Nutrition Facts added sugar labeling policy in the US by age, gender, and race/ethnicity over a lifetime

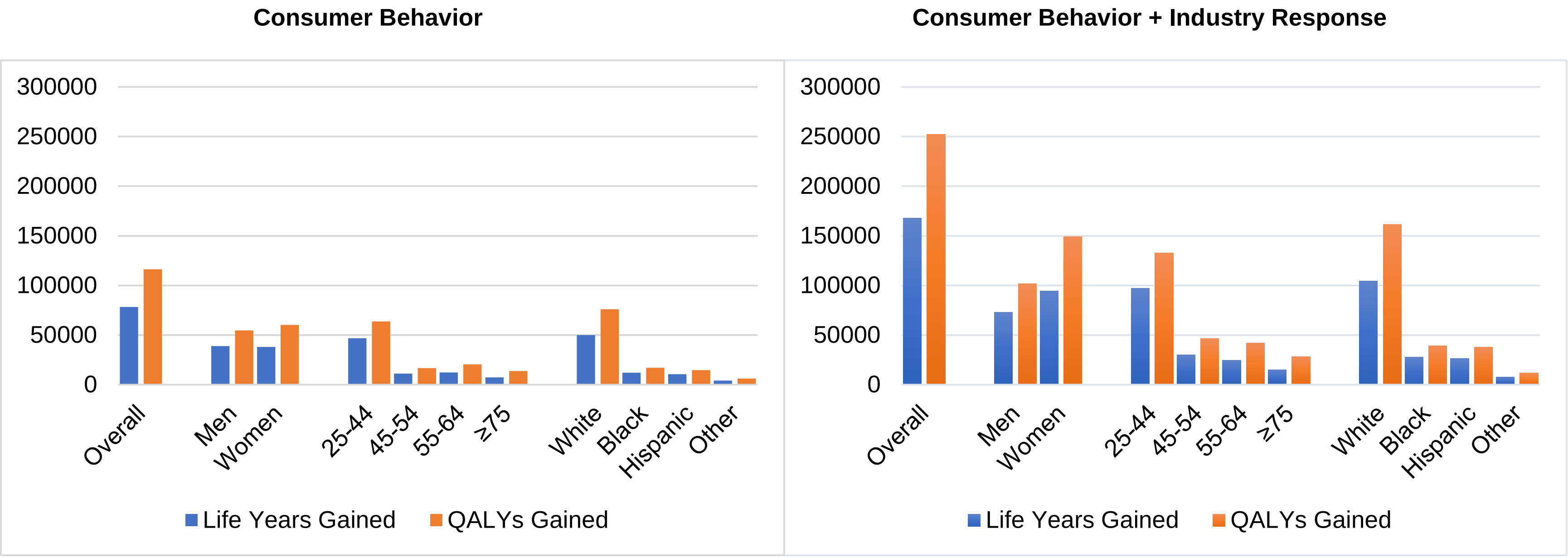

© 2021 Du M et al. *JAMA Network Open.*

eFigure 4. Estimated changes of heath-related costs associated with Nutrition Facts added sugar labeling policy in the US by age, gender, race/ethnicity and cancer type over a lifetime

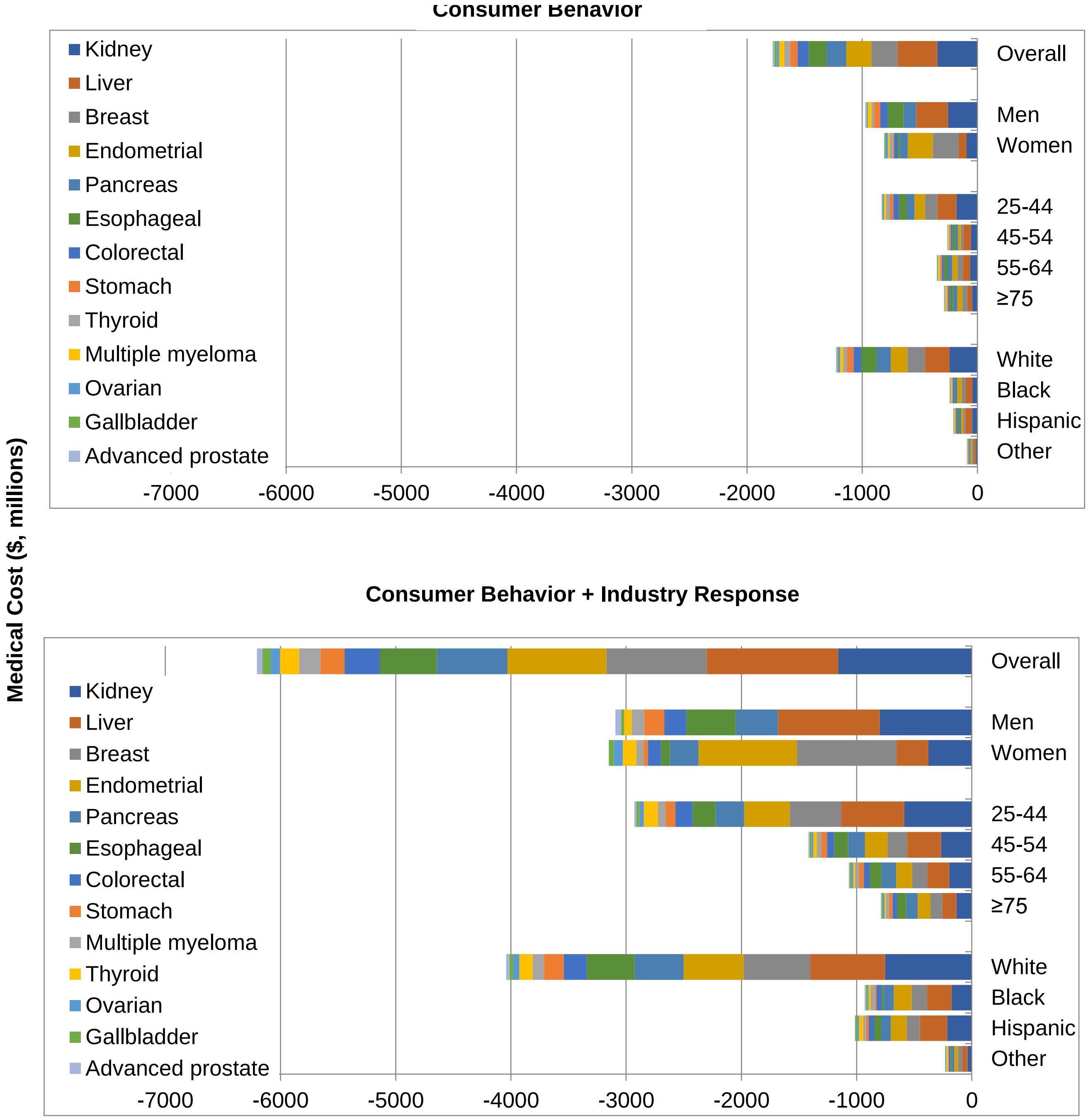

(A)

© 2021 Du M et al. *JAMA Network Open.*

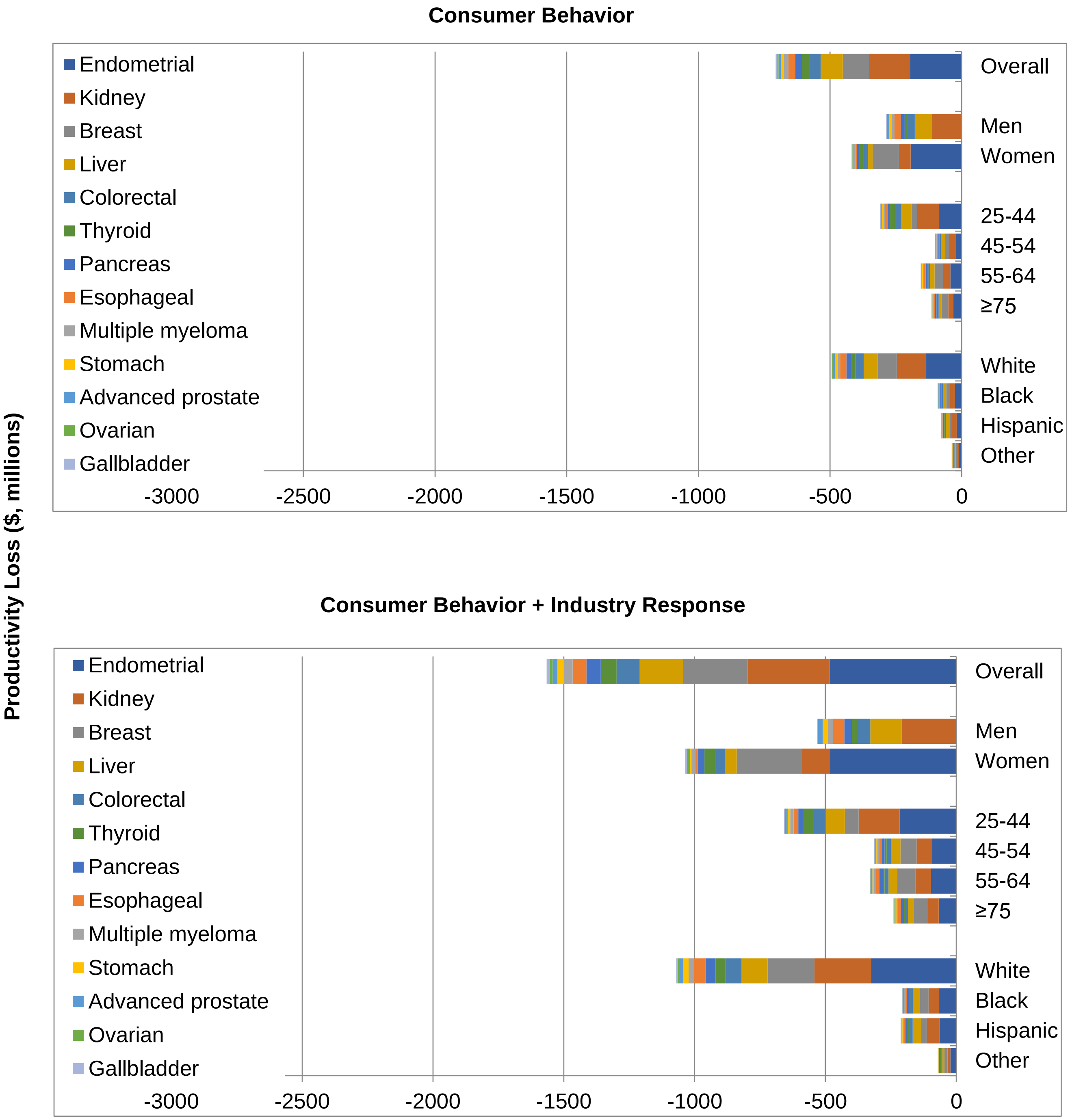

(B)

© 2021 Du M et al. *JAMA Network Open.*

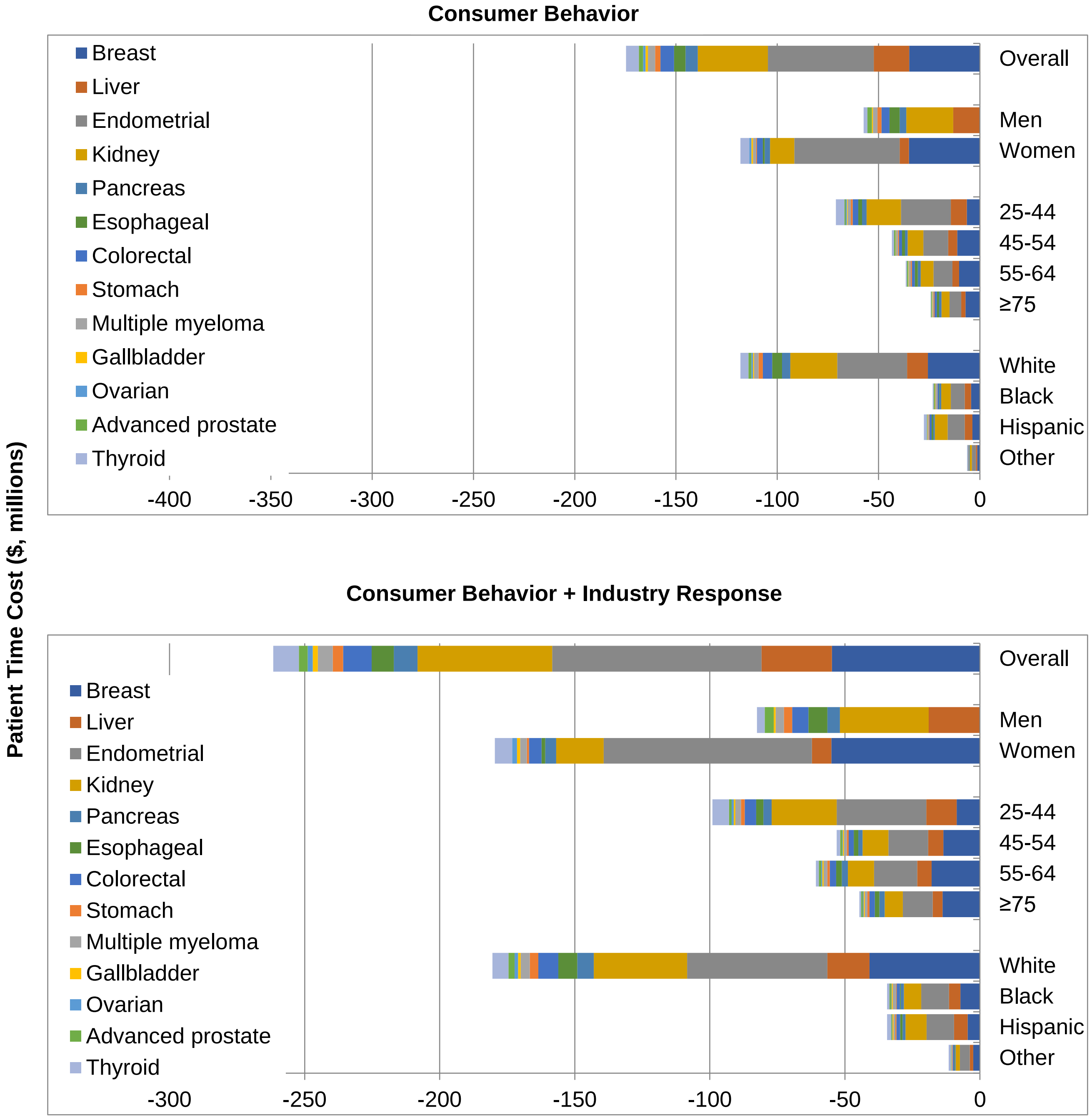

(C)

© 2021 Du M et al. *JAMA Network Open.*

eFigure 5. Estimated net costs from societal and healthcare perspectives associated with Nutrition Facts added sugar labeling policy in the US by age, gender, and race/ethnicity over a lifetime

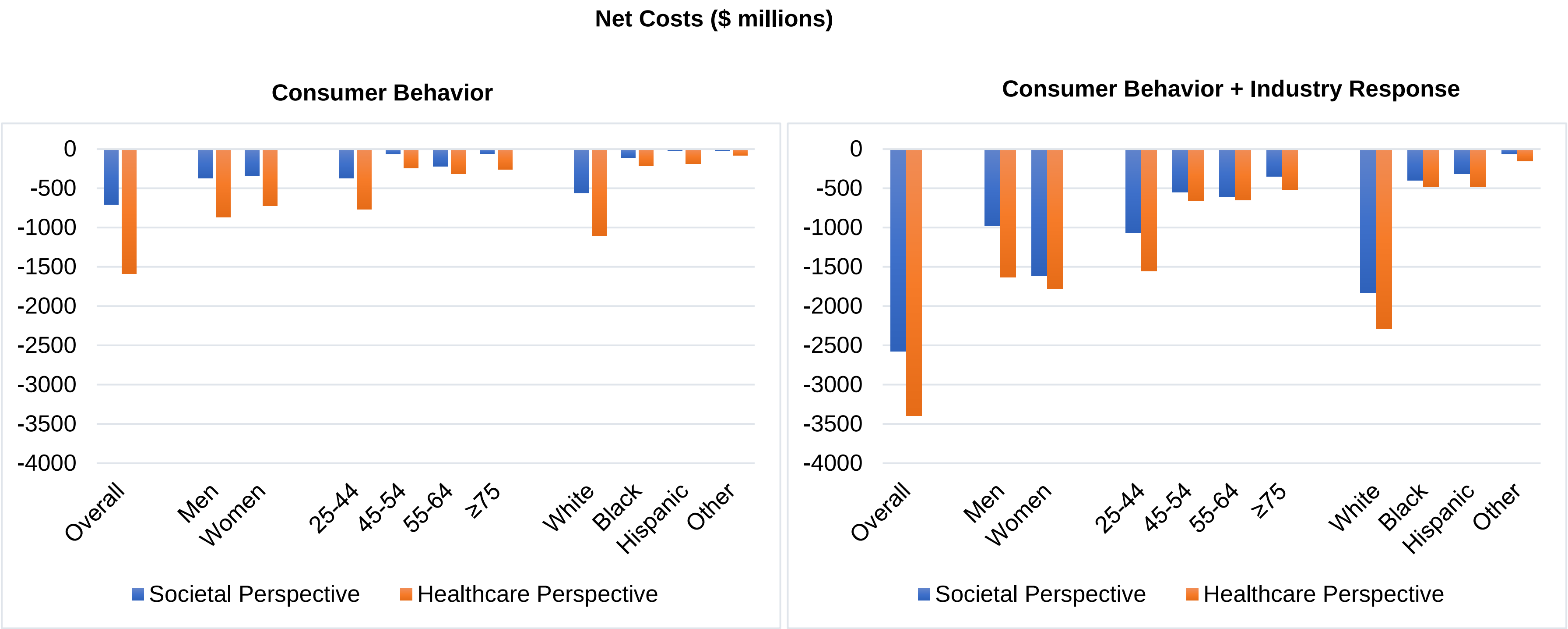

© 2021 Du M et al. *JAMA Network Open.*

eFigure 6. One-way sensitivity analysis of net costs of Nutrition Facts added sugar label and obesity-associated cancer rates to varying assumptions of key input parameters from (A) societal perspective and (B) healthcare perspective

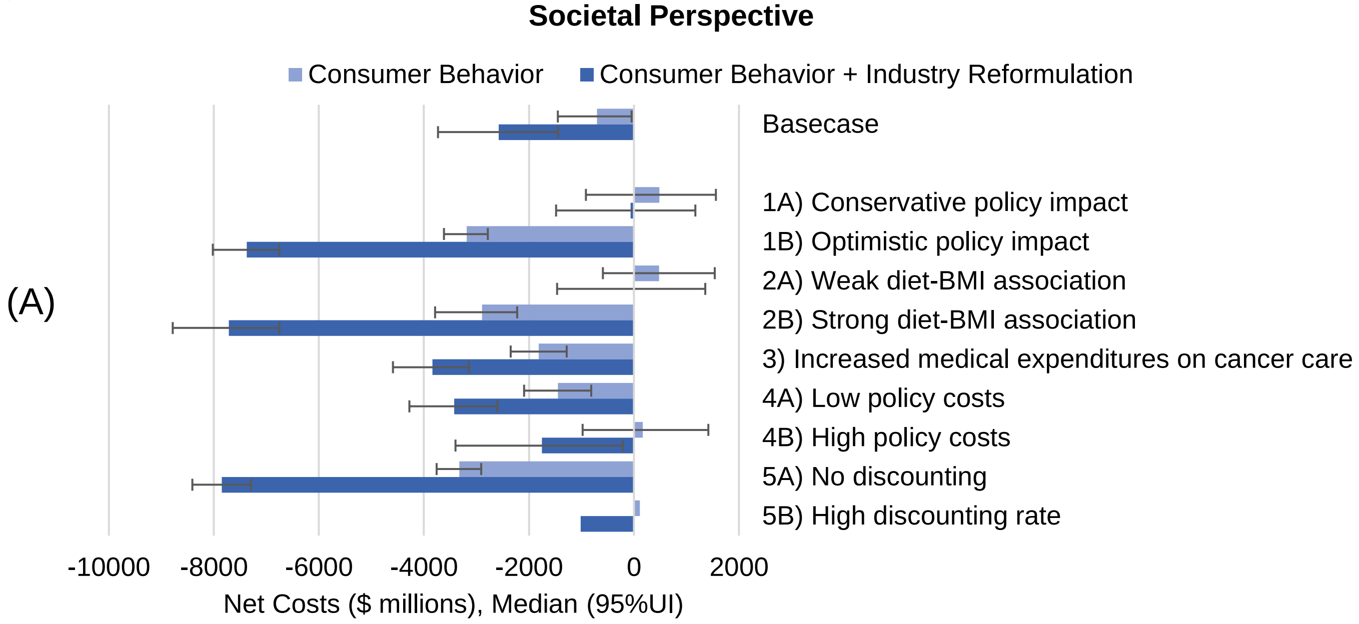

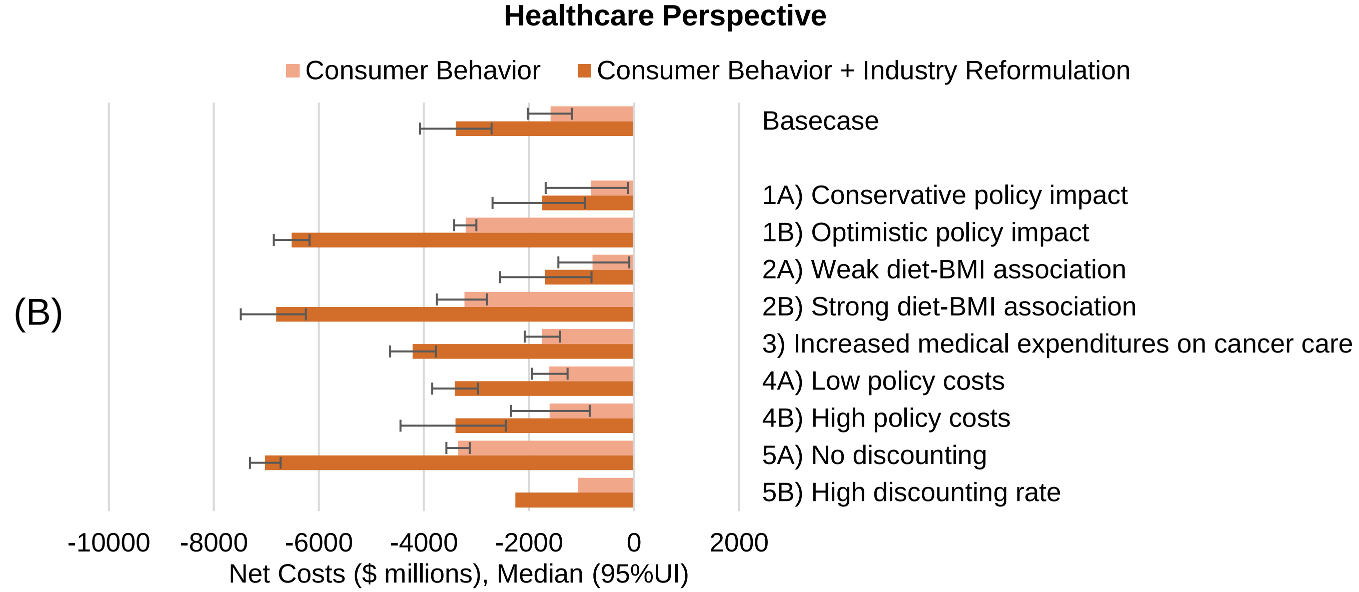

1a) conservative policy impact assumed half of the base-case policy impact (consumer behavior: -3.3%; industry reformulation: -4.13%); 1b) optimistic policy impact assumed two times of the base-case policy effect (consumer behavior: -13.0%; industry reformulation: -16.5%); 2a) weaker diet-BMI association assumed half of the base-case diet-BMI association (healthy-weight: 0.05 kg/m^2; overweight/obese: 0.12 kg/m^2); 2b) stronger diet-BMI association assumed two times of the base-case diet-BMI association (healthy-weight: 0.20 kg/m^2; overweight/obese: 0.46 kg/m^2); 3) 2% annual increase in medical expenditure on cancer care; 4a) lower policy implementation costs assumed 25% of the total costs for implementing the Nutrition Facts label; 4b) higher policy implementation costs assumed 75% of the total costs for implementing the Nutrition Facts label; 5a) lower discounting rate assumed 0% discounting rate; and 5b) higher discounting rate assumed 5% discounting rate. Under base-case scenario (policy effect assumed consumer behavior: -6.6%, and industry reformulation: -8.25%; diet-BMI association assumed healthy-weight: 0.1 kg/m2, and overweight/obese: 0.23 kg/m2; medical expenditure on cancer care assumed 0% annual increase; policy implementation costs assumed 50% of the total costs for implementing the Nutrition Facts label; discounting rate assumed 3%), the policy was cost-saving from both societal and healthcare perspectives. The policy remained cost-saving for all sensitivity analyses from the healthcare perspective and from societal perspective with additional industry reformulation. With consumer behavior alone, the policy was cost-saving when under 1b, 2b, 3, 4a, and 5b and was cost-effective under 1a (ICER: $8330/QALY), 2a ($8320/QALY), 4b ($933/QALY), and 5b ($1680/QALY).

© 2021 Du M et al. *JAMA Network Open.*

eReferences

1. Centers for Disease Control and Prevention. NPCR and SEER Incidence – U.S. Cancer Statistics Public Use Databases. United States Department of Health and Human Services, Centers for Disease Control and Prevention and National Cancer Institute. www.cdc.gov/cancer/uscs/public-use. Accessed2018.
2. Mariotto AB, Yabroff KR, Shao Y, Feuer EJ, Brown ML. Projections of the cost of cancer care in the United States: 2010-2020. *Journal of the National Cancer Institute.* 2011;103(2):117-128.
3. Clegg LX, Hankey BF, Tiwari R, Feuer EJ, Edwards BK. Estimating average annual per cent change in trend analysis. *Statistics in medicine.* 2009;28(29):3670-3682.
4. United States Census Bureau. Projections for the United States: 2017 to 2060. https://www.census.gov/data/tables/2017/demo/popproj/2017-summary-tables.html. Published 2018. Accessed.
5. National Cancer Institute. Surveillance research Program. Measures of Cancer Survival. https://surveillance.cancer.gov/survival/measures.html. Accessed.
6. Brenner H, Hakulinen T. Up-to-date and precise estimates of cancer patient survival: model-based period analysis. *American journal of epidemiology.* 2006;164(7):689-696.
7. Brenner H, Hakulinen T. Up-to-date cancer survival: period analysis and beyond. *International journal of cancer.* 2009;124(6):1384-1390.
8. National Cancer Institute. Surveillance Research Program. Cancer Survival Statistics: Cohort Definition Using Diagnosis Year. https://surveillance.cancer.gov/survival/cohort.html. Accessed.
9. Shangguan S, Afshin A, Shulkin M, et al. A Meta-Analysis of Food Labeling Effects on Consumer Diet Behaviors and Industry Practices. *American journal of preventive medicine.* 2019;56(2):300-314.
10. Huang Y, Kypridemos C, Liu J, et al. Cost-Effectiveness of the US Food and Drug Administration Added Sugar Labeling Policy for Improving Diet and Health. *Circulation.* 2019;139(23):2613-2624.
11. Food and Drug Administration. Regulatory impact analysis for final rules on "Food labeling: revision of the nutrition and supplement facts label". In: Food and Drug Administration, ed2017.
12. Public Health England. *Sugar Reduction, achieving the 20%: A technical report outlining progress to date, guidelines for industry, 2015 baseline levels in key foods and next steps.* 2017.
13. Food and Drug Administration. *Justification of Estimates for Appropriations Committees Fiscal Year 2012.* 2012.
14. Food and Drug Administration. *The Nutrition Review Project. Report to the Director, Center for Food Safety and Applied Nutrition.* 2014.
15. Sanders GD, Neumann PJ, Basu A, et al. Recommendations for Conduct, Methodological Practices, and Reporting of Cost-effectiveness Analyses: Second Panel on Cost-Effectiveness in Health and Medicine. *Jama.* 2016;316(10):1093-1103.
16. Guy GP, Jr., Ekwueme DU, Yabroff KR, et al. Economic burden of cancer survivorship among adults in the United States. *Journal of clinical oncology : official journal of the American Society of Clinical Oncology.* 2013;31(30):3749-3757.
17. Hogan C, Lunney J, Gabel J, Lynn J. Medicare beneficiaries' costs of care in the last year of life. *Health affairs (Project Hope).* 2001;20(4):188-195.
18. Yabroff KR, Davis WW, Lamont EB, et al. Patient time costs associated with cancer care. *Journal of the National Cancer Institute.* 2007;99(1):14-23.
19. Yabroff KR, Guy GP, Jr., Ekwueme DU, et al. Annual patient time costs associated with medical care among cancer survivors in the United States. *Medical care.* 2014;52(7):594-601.
20. Zheng Z, Yabroff KR, Guy GP, Jr., et al. Annual Medical Expenditure and Productivity Loss Among Colorectal, Female Breast, and Prostate Cancer Survivors in the United States. *Journal of the National Cancer Institute.* 2016;108(5).
21. Martin AB, Hartman M, Washington B, Catlin A. National Health Care Spending In 2017: Growth Slows To Post-Great Recession Rates; Share Of GDP Stabilizes. *Health affairs (Project Hope).* 2019;38(1):101377hlthaff201805085.
22. Lauby-Secretan B, Scoccianti C, Loomis D, Grosse Y, Bianchini F, Straif K. Body Fatness and Cancer--Viewpoint of the IARC Working Group. *The New England journal of medicine.* 2016;375(8):794-798.

© 2021 Du M et al. *JAMA Network Open.*

23. Naik H, Howell D, Su S, et al. EQ-5D Health Utility Scores: Data from a Comprehensive Canadian Cancer Centre. *The patient.* 2017;10(1):105-115.
24. Wildi SM, Cox MH, Clark LL, et al. Assessment of health state utilities and quality of life in patients with malignant esophageal Dysphagia. *The American journal of gastroenterology.* 2004;99(6):1044-1049.
25. Pickard AS, Jiang R, Lin HW, Rosenbloom S, Cella D. Using Patient-reported Outcomes to Compare Relative Burden of Cancer: EQ-5D and Functional Assessment of Cancer Therapy-General in Eleven Types of Cancer. *Clinical therapeutics.* 2016;38(4):769-777.
26. Zhou HJ, So JB, Yong WP, et al. Validation of the functional assessment of cancer therapy-gastric module for the Chinese population. *Health and quality of life outcomes.* 2012;10:145.
27. Yabroff KR, McNeel TS, Waldron WR, et al. Health limitations and quality of life associated with cancer and other chronic diseases by phase of care. *Medical care.* 2007;45(7):629-637.
28. Muller-Nordhorn J, Roll S, Bohmig M, et al. Health-related quality of life in patients with pancreatic cancer. *Digestion.* 2006;74(2):118-125.
29. Farkkila N, Sintonen H, Saarto T, et al. Health-related quality of life in colorectal cancer. *Colorectal disease : the official journal of the Association of Coloproctology of Great Britain and Ireland.* 2013;15(5):e215-222.

© 2021 Du M et al. *JAMA Network Open.*
